# Compatibility and Stability of a *Shigella* Polysaccharide—Protein Conjugate Antigen Formulated with Aluminum Salt and CpG 1018^®^ Adjuvants

**DOI:** 10.3390/vaccines14010010

**Published:** 2025-12-20

**Authors:** Poorva Taskar, Prashant Kumar, Brandy Dotson, Anup Datta, Shangdong Guo, Giriraj Chalke, Richa Puri, Harshita Seth, Benjamin Wizel, Sangeeta B. Joshi, David B. Volkin

**Affiliations:** 1 Vaccine Analytics and Formulation Center, Department of Pharmaceutical Chemistry, University of Kansas, Lawrence, KS 66047, USA; 2 Inventprise Inc., Redmond, WA 98052, USA; 3 Dynavax Technologies Corporation, 2929 Seventh Street, Suite 100, Berkeley, CA 94710, USA

**Keywords:** *Shigella* vaccine, polysaccharide–protein conjugate vaccine, compatibility, stability, formulation, adjuvant, aluminum salt, CpG 1018, ELISA

## Abstract

This study evaluated the formulation and stability of a quadrivalent glycoconjugate *Shigella* vaccine candidate based on four predominant strains (*S. flexneri*; *2a*, *3a*, and *6*, and *S. sonnei*) covering ~64% of global *Shigella* infections. Each glycoconjugate antigen consists of a strain-specific O-polysaccharide (O-PS) covalently linked to the carrier protein IpaB, a component of the *Shigella* type III secretion system. First, selective competitive ELISAs were developed to measure antigenicity of the four O-PS-IpaB conjugates formulated with different adjuvants (i.e., Alhydrogel^®^, AH; Adju-phos^®^, AP; and CpG-1018^®^, CpG). Next, the monovalent *S. sonnei* O-PS-IpaB conjugate was studied to elucidate interactions with aluminum salt adjuvants (AH, AP) under different solution conditions. Third, the stability profiles of AH- or AP-adjuvanted *S. sonnei* O-PS-IpaB conjugate in various formulations (±CpG) were determined at different temperatures. Interestingly, incubation at 25 °C for 2 weeks resulted in increased antigenicity values when the antigen was bound to AP or AH, suggesting increased epitope exposure upon adjuvant binding. When bound to AP adjuvant at pH 5.8, the best glycoconjugate antigen stability was observed at elevated temperatures. The CpG adjuvant under these conditions, however, displayed incompatibility (i.e., material loss), presumably from precipitation due to lack of interaction with AP and presence of the detergent LDAO from the bulk antigen buffer. In contrast, the glycoconjugate antigen and CpG adjuvant were both bound to the AH adjuvant and stable at 2–8 °C, pH 7.0. This AH-CpG formulation of the O-PS-IpaB conjugate antigens was identified as a promising candidate for future animal immunogenicity testing.

## 1. Introduction

Shigellosis is an acute gastrointestinal disease caused by the gram-negative bacterium *Shigella*. The genus *Shigella* is classified into four species: *S. dysenteriae* (also referred to as group A), *S. flexneri* (group B), *S. boydii* (group C), and *S. sonnei* (group D), with each species having multiple serotypes, approximately 50 strains across all groups [1,2]. Shigellosis is mainly caused by *S. sonnei* in high-income countries (HICs) and *S. flexneri* in low-and-middle-income countries (LMICs). *Shigella* transmission occurs through the fecal–oral route, via contaminated water and food [1], primarily due to lack of access to clean water and poor sanitation [2,3]. Children under the age of 5 in LMICs are at the highest risk, with Shigellosis being the primary cause for moderate to severe diarrhea in the second year of life [4]. Shigellosis accounts for ~13% of global diarrheal mortality, and every year, an estimated higher range of 80–165 million cases of disease and 600,000 deaths among children [5,6].

Shigellosis is often treated with antibiotics, causing an increase in antimicrobial-resistant strains, and in some cases, extensively drug-resistant *Shigella* strains [6,7]. For example, antibiotic-resistant *Shigella* strains have risen in the USA from nearly 0% in 2015 to 5% in 2022 [7], with similar increasing trends observed globally. Notably, *S. flexneri* and *S. sonnei* strains have demonstrated resistance to commonly used antibiotics, including azithromycin, ciprofloxacin, ceftriaxone, trimethoprim-sulfamethoxazole, and ampicillin [7,8,9]. In response, the World Health Organization (WHO) now recommends restricting antibiotic treatment for non-invasive diarrhea to reduce selective pressure and slow the spread of resistant strains [10]. Although new antibiotics are needed to treat drug-resistant *Shigella* strains, they will eventually face the same trajectory of causing new drug-resistant strains [11]. To address the unmet medical needs of combating the high global health burdens of shigellosis and mitigating the rise in antibiotic-resistant strains, [1,6] a safe and effective *Shigella* vaccine is urgently needed, yet currently there are no licensed, commercially available vaccines for *Shigella*. For these reasons, the WHO lists *Shigella* as a priority pathogen of public health importance for vaccine development [6].

Unfortunately, progress in *Shigella* vaccine development has been scientifically challenging even after a century of R&D efforts [12,13]. The complexity of both the pathogen and the disease, including the large number of diverse serotypes, has created hurdles for identifying a broadly protective vaccine candidate. For example, prior studies have provided strong evidence that vaccine-induced protection against Shigellosis is serotype-specific; therefore, a vaccine candidate must contain a mixture of multivalent *Shigella* antigens [1,12]. In fact, the presence of the pathogen’s multiple serotypes and sub-serotypes has been a significant hurdle for developing an effective *Shigella* vaccine candidate. Moreover, since Shigellosis causes the highest disease burden in LMICs, investments in vaccine R&D face significant challenges due to limited funding and the lack of commercial incentives [2]. *Shigella* vaccine development approaches have explored both traditional vaccine platforms (i.e., inactivated, live attenuated approaches) as well as recombinant subunit platforms (i.e., glycoconjugates prepared with novel formulation strategies including different carrier proteins and adjuvants) as described below.

Although traditional vaccine platforms, including inactivated and live attenuated vaccines, have shown some promise during initial studies, many candidates have been discontinued due to high reactogenicity and/or insufficient efficacy. Additionally, environmental enteric dysfunction (EED), commonly observed in children in LMICs, has negatively affected the studies with oral live attenuated vaccine candidates, ultimately leading to discontinuation [12]. Despite these challenges, some recent progress has been made with live attenuated *Shigella* vaccine candidates, including (1) WRSs2 and WRSs3, which are monovalent *S. sonnei* candidates developed by Walter Reed Army Institute of Research (WRAIR) and have completed Phase 1 clinical studies [14], and (2) a combined Shig-ETEC vaccine by Eveliqure, comprising live attenuated *S. flexneri 2a* engineered to express toxins of enterotoxigenic *E. coli* that is scheduled to start Phase 2 clinical studies [12,13,15].

The subunit vaccine platform is being utilized by many of the currently active *Shigella* preclinical and clinical vaccine development programs, with glycoconjugates being the most common type. This strategy is based on the key antigenic determinant, the O-antigen, which is a repetitive glycan polymer on the surface of the bacteria [12]. However, the O-antigen polysaccharide (O-PS) alone generates a T-cell independent immune response, and thus directly triggers B-cell activation with production of short-lived and lower-affinity antibody production [16]. To overcome this limitation, a carrier protein is conjugated to O-PS, allowing the polysaccharide to be expressed on the major histocompatibility complex class II (MHC II) receptor, resulting in more robust immune responses including immunological memory as well as cell-mediated and humoral immunity [16]. Examples of glycoconjugate and subunit antigens in clinical development of *Shigella* vaccine candidates include (1) a bivalent conjugate vaccine candidate (ZF0901) made by Beijing Zhifei Lvzhu Biopharmaceuticals, comprising *S. flexneri 2a* and *S. sonnei* O-PS conjugated to tetanus toxoid (Phase 2 clinical study) [12], (2) LimmaTech Biologics which has produced glycoconjugates in vivo in bacterial cells [17] and developed S4V-EPA, a vaccine candidate comprising O-PS from *S. flexneri 2a, 3a, 6, and S. sonnei* conjugated to detoxified exoprotein A of *Pseudomonas aeruginosa* (rEPA) (Phase 2 trial), [12,17] (3) Invaplex_AR_ by WRAIR which is based on a *S. flexneri* 2a lipopolysaccharide (LPS) complexed with conserved Invasion plasmid antigen B and C (i.e., IpaB and IpaC) proteins (Phase 1 clinical study), (4) altSonflex1-2-3 candidate made by GSK based on Quadrivalent *S. flexneri 1b, 2a, 3a, and S. sonnei* native outer membrane vesicles (Phase 2 clinical trial), and (5) SF2a-TT15 by Pasteur Institute, based on synthetically prepared *S. flexneri* 2a synthetic O-antigen, as a glycoconjugate antigen (Phase 2 clinical study) [12,13].

In this work, the antigenic component of *Shigella* glycoconjugate vaccine candidate comprises four polysaccharide strains, three *S. flexneri* (*S. f.*) serotypes: *S. f. 2a, S. f. 3a, S. f. 6*, and *S. sonnei,* each conjugated to IpaB as a carrier protein. The Global Enteric Multicenter Study (GEMS) found that a vaccine candidate containing the O-antigens of *Shigella flexneri 2a, 3a, 6*, and *Shigella sonnei* could directly protect against 64% of global *Shigella* isolates, and with cross-protection against other flexneri serotypes, could extend coverage to as much as 88% [18]. The carrier protein, IpaB, is a virulence factor of *Shigella* type III secretion system and is highly conserved among multiple serotypes [13,19]. In addition to serving as a carrier protein for the four O-PS antigens, the IpaB protein may also serve as a vaccine antigen. For example, there is interest in developing the IpaB protein, fused with other *Shigella* and immunostimulatory proteins, as a subunit vaccine candidate [20]. A monovalent *S. f. 2a* O-PS-IpaB glycoconjugate vaccine candidate has been prepared and showed broad protection against multiple serotypes in mice [19], a promising result for this vaccine design approach. Moreover, in a separate study, a quadrivalent mixture of O-PS-IpaB conjugate antigens (with the same four O-PS used in this study) displayed promising immunogenicity results in rabbits that infer the IpaB carrier protein, generating some cross-serotype protection [21].

The goal of this work was to evaluate the feasibility of developing a low-cost, adjuvanted formulation of this *Shigella* glycoconjugate vaccine candidate targeted for use in LMICs. To this end, O-PS-IpaB vaccine antigens were formulated with two commonly used adjuvants, aluminum salts (Alhydrogel^®^, AH; Adju-phos^®^, AP) and CpG (CpG-1018^®^), either individually or in a combination, and their interactions, compatibility, and stability profiles were assessed. We developed a series of competitive ELISAs to quantify these antigens both free in solution and upon being adsorbed to aluminum salt adjuvants (AH, AP) in the presence and absence of the CpG adjuvant. The key findings are discussed, along with lessons learned and recommendations for future analytical and formulation development work with this *Shigella* protein–polysaccharide vaccine candidate.

## 2. Materials and Methods

### 2.1. Materials

Four different serotype-specific *Shigella* O-polysaccharides (O-PSs), *Shigella flexneri 2a* (*S. f. 2a*), *Shigella flexneri 3a* (*S. f. 3a*), *Shigella flexneri 6* (*S. f. 6*), and *Shigella sonnei* (*S. sonnei*), were manufactured by Inventprise (Redmond, WA, USA). The O-PSs were provided in milli-Q water and were individually aliquoted under aseptic conditions into 1.5 mL Eppendorf tubes and stored at −80 °C before use. The carrier protein IpaB was manufactured by Inventprise (Redmond, WA, USA). Material was received in a buffer containing 20 mM Tris, 0.5 M sodium chloride (NaCl), and 0.2% w/v lauryl dimethylamine oxide (LDAO) at pH 7.5–7.9 and was similarly aliquoted as described above and stored at −80 °C.

The four different O-PS-IpaB conjugate antigens were prepared by chemical conjugation using 1-cyano-4-dimethylaminopyridine tetrafluoroborate (CDAP) by Inventprise (Redmond, WA, USA) with the CDAP conjugation process described elsewhere [22]. The antigens were received in a buffer comprising 20 mM sodium phosphate, 0.15 M NaCl, 0.05% (w/v) LDAO, and pH 7–7.5. Each of the four different O-PS-IpaB conjugate antigens was similarly aliquoted as described above and stored at 2–8 °C before use.

Two different aluminum salt adjuvants: Alhydrogel^®^ (AH, #vac-alu-250), and Adju-phos^®^ (AP, #vac-phos-250), were purchased from InvivoGen (San Diego, CA, USA). The oligonucleotide adjuvant, cytosine-phosphate-guanine 1018 (CpG 1018^®^), was provided by Dynavax (Berkeley, CA, USA). Antibody reagents for the competitive ELISAs are described below. Sodium phosphate monobasic monohydrate(#S369-500), sodium phosphate dibasic heptahydrate (#S373-500), and sodium chloride (#S640-500) were purchased from Fisher Scientific (Waltham, MA, USA). Lauryldimethylamine-N-oxide (#A65504) was purchased from Thermo Fisher Scientific (Waltham, MA, USA).

### 2.2. Methods

#### 2.2.1. Preparation of Formulated Samples of O-PS-IpaB Conjugate Antigens with Adjuvants

The formulated samples were prepared aseptically in a biosafety hood by diluting stock solutions of *Shigella* O-PS-IpaB conjugates to 30 µg/mL (2X antigen) in a buffer containing 2 or 200 mM sodium phosphate, 0.15 M NaCl, 0.0 or 0.05% w/v LDAO at pH 7.0 or 5.8. Samples were mixed for ~20 min. A 2X adjuvant stock solution in the same buffer was prepared by diluting the aluminum salt adjuvants (10 mg/mL for AH and 5 mg/mL for AP) and/or CpG 1018^®^ stock solution (12 mg/mL) to 1.5 mg/mL of AH or AP, and/or 0.6 mg/mL of CpG, and mixed to ensure binding of CpG to the aluminum salt adjuvants. The final formulated sample was prepared by mixing equal volumes of the 2X antigen solution and 2X adjuvant stock for final concentration of 15 µg/mL *Shigella* conjugate(s), 0.75 mg/mL AH or AP, and/or 0.3 mg/mL CpG in 2 or 200 mM sodium phosphate, 0.15 M NaCl, 0.004% (quadrivalent) or 0.001% (monovalent) or 0.05% w/v LDAO at pH 7.0 or 5.8. The final solution pH was adjusted to 7.0 or 5.8 using 0.1 N NaOH or 0.1 N HCl and mixed for 2 h at room temperature followed by 2–8 °C storage overnight.

#### 2.2.2. Competitive ELISAs for *Shigella* O-PS-IpaB Conjugates

The competitive ELISA was developed to measure antibody binding to vaccine antigen (i.e., antigenicity) in the presence and absence of aluminum salt adjuvants (AH or AP). The primary antibody reagents for each *Shigella* O-PS-IpaB conjugate were provided by Inventprise (Redmond, WA, USA) and each was specific for the O-polysaccharide component of the conjugate vaccine. Primary antibodies against O-PS for *S. f. 3a* and *S. f. 6* were rabbit polyclonal antibodies (pAb), while those for *S. f. 2a* and *S. sonnei* were mouse monoclonal antibodies (mAb). Secondary detection antibodies were purchased from either Millipore (Goat-anti-mouse IgG HRP pAb, Burlington, MA, USA) or Invitrogen (Goat-anti-rabbit IgG HRP pAb, Carlsbad, CA, USA).

The competitive ELISA format was adapted from a procedure described elsewhere [23,24] for different vaccine antigens. Aluminum salt adjuvanted or unadjuvanted antigen samples were analyzed at 5 µg/mL (a three-fold dilution for each antigen prepared at 15 µg/mL) by serially diluting across a 96-well plate and adding an excess known concentration of primary antibody and incubating overnight at room temperature. The plate was centrifuged at 1500× *g* for 5 min, and the supernatant containing unbound primary antibody was transferred to 96-well microplates that had been coated with the O-PS-IpaB antigen overnight at 2–8 °C. The primary antibody was detected by a commercial secondary antibody conjugated to horseradish peroxidase (HRP) that interacts with tetramethylbenzidine (TMB) and was analyzed at 450 nm once the reaction was stopped using 1 M sulfuric acid. A 4-PL fit was used for the data analysis in Origin 2020^®^ software (Origin lab corporation, Wellesley, MA, USA) using inverse prediction method.

#### 2.2.3. Protein Concentration of *Shigella* O-PS-IpaB Conjugate Antigens Using Micro-Bicinchoninic Acid Assay (m-BCA)

The protein concentration of antigens was determined by an m-BCA procedure [25] in the presence and absence of aluminum salt adjuvants (AH and AP). The Micro BCA™ Protein Assay Kit (ThermoFisher Scientific, Catalog# 23235, Waltham, MA, USA) included three reagents: an alkalizing solution (MA), a bicinchoninic acid solution (MB), and a copper sulphate solution (MC). Briefly, 150 µL of supernatant was transferred to a 96-well microplate. Equal volume of m-BCA reagent mixture (25:24:1 MA: MB: MC) was added to the supernatant in the microplate. Quantification was performed by a polynomial fit standard curve of BSA from 200 to 0.5 µg/mL. The plate was sealed and incubated for 2 h at 37 °C resulting in development of purple color, proportional to the protein in the sample, was detected at 562 nm using a plate reader.

#### 2.2.4. CpG 1018 Concentration Using Ultraviolet-Visible (UV-Vis) Spectroscopy

CpG 1018^®^ concentration was measured by A260 nm values in the presence and absence of aluminum salt adjuvants using an adapted procedure from a previous report [26]. Briefly, NanoDrop™ Spectrophotometer 2000 (ThermoFisher Scientific, Waltham, MA, USA) was used for UV analysis. First, the measuring surface was cleaned with distilled water, and a blank measurement of 2.0 µL of the buffer present in the respective formulations was performed. Then, a 2.0 µL sample of the supernatant was added, and the A260 nm values were recorded. To determine the concentration of CpG, an extinction coefficient of (25.6 mL mg^−1^ cm^−1^ [27]) was used.

#### 2.2.5. Binding Studies Between Aluminum Salt Adjuvants and Either O-PS-IpaB Conjugate Antigens or CpG Adjuvant

To determine the adsorption of *Shigella* O-PS-IpaB conjugate antigens to aluminum salt adjuvants, 0.5 mL of aluminum salt adjuvant-adsorbed antigen was centrifuged at 15,000× *g* for 5 min in a microcentrifuge tube, and 0.45 mL supernatant was removed. The supernatant fraction and a control antigen sample (prepared in same way without adjuvant) were analyzed by m-BCA assay. The percentage of antigen adsorption to the adjuvant was expressed as follows:

(amount of conjugate in control − amount of conjugate in supernatant)/amount of conjugate in control) × 100.

The degree of CpG adjuvant adsorption to aluminum salt adjuvants was measured after separation of supernatant and pellet fractions as described above. The supernatant fraction and a control CpG sample (prepared in same way without aluminum salt adjuvant) were each analyzed using the CpG spectroscopic assay (described above). The percentage CpG adjuvant adsorption to the aluminum salt adjuvant was expressed as follows:

(amount of CpG in control sample − amount of unbound CpG in the supernatant)/amount of CpG in the control sample) × 100.

#### 2.2.6. Langmuir Isotherm Binding Studies

Studies to determine the adsorptive properties of *S. sonnei* O-PS-IpaB conjugate antigen to aluminum salt adjuvants in the presence and absence of CpG adjuvant were performed as follows: first, a fixed concentration of 40 µg/mL of aluminum salt adjuvants and 16 µg/mL of CpG were prepared by diluting the AH, AP, and CpG bulks to 1 mg/mL in a base solution of 0.15 M NaCl, 0.05% w/v LDAO at pH 7.0 (AH-containing formulations) or pH 5.8 (AP-containing formulations). The antigen concentration varied from 10 to 70 µg/mL by dilution from the stock solution. A 0.25 M sodium phosphate buffer was also prepared at pH 7.0 and 5.8 and was added to each sample considering carryover phosphate from the antigen bulk for the final phosphate concentration to be 2 mM.

The order of addition was: (1) aluminum salt adjuvant, (2) phosphate buffer, (3) CpG, and (4) antigen. The samples were mixed for 2 h at room temperature and stored at 2–8 °C overnight before centrifuging at 15,000× *g* for 10 min and analyzing the supernatant using m-BCA (see Section 2.2.3 above) to measure the protein concentration in the supernatant (*C_e_*). The binding data were plotted using the Langmuir equation (left) and the linearized form of the equation (right) [28], as shown below:

$$q_{e}=\frac{Q_{max}K_{L}C_{e}}{1+K_{L}C_{e}}\;\frac{C_{e}}{q_{e}}=\frac{1}{Q_{max}}C_{e}+\frac{1}{K_{L}Q_{max}}$$

The adsorptive properties including maximum monolayer binding capacity (*Q_max_*) and strength of interaction (*K_L_*) were calculated using a linearized form of the Langmuir equation where *q_e_* is solid phase equilibrium concentration and *C_e_* is liquid phase equilibrium concentration.

#### 2.2.7. Zeta Potential of Aluminum Salt Adjuvants (AH, AP)

Zeta potential values of aluminum salt adjuvants in different formulations were measured using a ZetaPALS (Brookhaven Instruments Corporation, Nashua, NH, USA, NanoBrook 90Plus PALS) along with a solvent-resistant electrode (Brookhaven Instruments Corporation, Nashua, NH, USA, BI-SREL, SR-0338) conditioned with 1.5 mL of 0.9% w/v saline solution prepared in deionized water and measured using a cuvette. The criterion for proceeding with the experiment was for conductance to be >30,000 µS. The samples contained 1.5 mg/mL AH or AP, in the presence and absence of CpG (0.6 mg/mL), in a range of phosphate buffer (2–200 mM) and sodium chloride (0–1 M) concentrations, with 0.05% w/v LDAO at pH 7.0 or 5.8. For the experiment, 15 µL of the sample was diluted to 1.5 mL with deionized water in a cuvette. The experiment was run at room temperature with ten cycles and equilibrated for 180 s.

#### 2.2.8. Isoelectric Point (pI) Estimations Using Spin Columns

The isoelectric point (pI) value of *S. sonnei* O-PS-IpaB conjugate was determined using cation exchange spin columns (ThermoFisher Scientific, catalog# 90008). These spin columns are hydrophilic and use a highly porous, cellulose-based membrane adsorption technique [29] and have sulfonic groups that bind to positively charged molecules. The pI value was determined using an adapted procedure from ThermoFisher Scientific; i.e., the antigen samples were prepared at pH 4.0 (pH < pI) to allow binding to the column and were eluted using a series of higher pH solutions (antigen elutes when pH ≥ pI) [29].

The buffers used for this study were 20 mM Phosphate-Citrate at pH 4.0, 5.0, 5.5, 6.0, 6.5, 7.0, 7.6, and 20 mM Tris-HCl at pH 8 and 9. Samples were prepared by diluting the *S. sonnei* conjugate to 0.17 mg/mL in the pH 4.0 buffer. The column was equilibrated with pH 4 buffer and 300 µL of sample was added and centrifuged at 500× *g* for 5 min. The flow-through was collected and a pH 4 wash was performed to ensure the binding of antigen to the column. Consecutive elution from pH 5 to 9 was performed by adding 400 µL of each buffer followed by centrifuging at 500× *g* for 5 min and collecting each flow-through separately. A 0.5 M sodium chloride solution was used to elute the remaining antigen on the column. The collected fractions were analyzed by m-BCA (see Section 2.2.3 above) using standard curves prepared for each buffer. The pH at which elution of antigen begins was identified as the pI of the antigen.

#### 2.2.9. Storage Stability Studies

Real-time (2–8 °C) and accelerated (25 and 37 °C) stability studies were carried out using monovalent formulations of *Shigella sonnei* O-PS-IpaB conjugate antigen in various formulations. As mentioned above, 2X stock solutions of monovalent *S. sonnei* were aseptically prepared in indicated buffers (see Section 3) and 2X adjuvant stocks comprising AH or AP and/or CpG were also aseptically prepared in the same indicated buffers. The two 2X stocks were mixed in a 1:1 ratio in 15 mL conical tubes, rotated for 2 h at room temperature and stored at 2–8 °C overnight to complete the adsorption of the antigen to the adjuvant.

The formulations were stored in pre-sterilized, ready-to-use 2 mL glass vials (Schott adaptiQ^®^, catalog#VIA RTU 0020 FC 13,00 EBB TL). The vials were stoppered using 13 mm Serum NovaPure^®^ rubber stoppers (#19700302) and were sealed using 13 mm Flip-off^®^ aluminum seals (#54131770, West Pharmaceutical, Exton, PA, USA). Once adsorbed, 0.6 mL of each formulation was added to each vial in the biosafety cabinet with intermittent mixing of the conical tube to ensure homogenous dispersion of aluminum salts in the formulations. Samples were incubated at 2–8 °C (3 weeks), 25 °C (2 weeks), and 37 °C (1 week) in the upright position and analyzed by competition ELISA (see Section 2.2.2 above).

#### 2.2.10. Thermal Stability Studies to Estimate Relative Degradation Profiles

The same *Shigella sonnei* O-PS-IpaB conjugate antigen formulations as described above were heat-stressed at temperatures ranging from 55 to 95 °C in increments of 5 °C by incubating in a water bath (Thermo Scientific, catalog #88870008). The temperature was initially set at 55 °C and aliquoted formulations were incubated at that temperature for 1 h after which the formulations were stored at 2–8 °C. The temperature of the water bath was then increased by 5 °C for the next batch of formulations to be stressed for 1 h, and this process was continued until all formulations were stressed at each of the indicated temperatures up to 95 °C. The formulations were filled under aseptic conditions and 110 µL was aliquoted in 1.5 mL epitubes. The heat-stressed samples were analyzed by competitive ELISA (see Section 2.2.2 above). The antigenicity values were determined, and each was compared to its control formulation incubated at 2–8 °C for 1 h. 

## 3. Results

### 3.1. Properties of Shigella OPS and O-PS-IpaB Conjugates and Adjuvants

**Figure 1:**
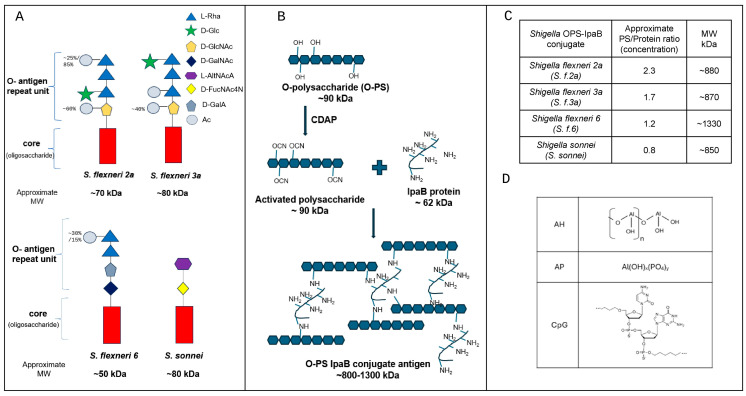
Structural schematic and experimental properties of the polysaccharide–protein conjugate *Shigella* antigens and adjuvants (aluminum salt and CpG oligonucleotide) used in this study. (**A**) Approximate size and chemical structure of each *Shigella* O-antigen polysaccharide (O-PS). (**B**) Schematic of the polysaccharide–protein conjugate produced by the 1-Cyano-4-Dimethylaminopyridine (CDAP) reaction via chemical conjugation of each *Shigella* O-PS to a carrier protein (*Shigella* invasion plasmid antigen B; IpaB). (**C**) Composition and approximate size ranges of the four *Shigella* O-PS-IpaB conjugates. [PS]—polysaccharide concentration measured by the Anthrone test. [Protein]—protein concentration measured by the BCA assay. MW—approx. molecular weight in kDa measured by SEC-HPLC. (**D**) Schematic structures of monomeric unit within the particulate adjuvants Alhydrogel^®^ (AH), Adju-phos^®^ (AP), and the oligonucleotide adjuvant CpG 1018^®^ (CpG). Panel A is adapted from Perepelov et al., 2012 [31], and Liu et al., 2008 [30], with permission from Oxford University Press. Panel B is adapted from Berti et al., 2018 [32], under the CC BY-NC 3.0 license. Panel D is adapted from Hickey et al., 2025 [33], under CC BY 4.0 license.

The schematic structures and properties of *Shigella* conjugate antigens and the aluminum salt and CpG 1018^®^ adjuvants used in this study are summarized in Figure 1. The *Shigella* polysaccharides comprise O-antigen repeating units attached to an oligosaccharide core. Each strain differs in the composition of the O-antigen repeating units: *S. f. 2a* and *S. f. 3a* have the same sugars in a different order, and *S. f. 6* and *S. sonnei* have sugars with acidic groups (Figure 1A) [30,31]. The *Shigella* O-PS-IpaB conjugate antigens were prepared by a random conjugation method with CDAP as a cyanylating reagent, which activates the hydroxyls present in polysaccharides, allowing them to react with amine groups in the carrier protein, IpaB (Figure 1B). The resulting structure of the polysaccharide–protein conjugate is large, complex, and variable [32]. The *Shigella* O-PS-IpaB conjugates have a polysaccharide to protein concentration ratio between 0.8 and 2.3 and an approximate size range of 800–1300 kDa (Figure 1C). The schematic structures of the monomeric units of the adjuvants evaluated in this work are shown in Figure 1D, in which the particulate adjuvants AH and AP consist of assemblies of aluminum salts comprising either hydroxyl groups or hydroxyl and phosphate groups, respectively [33]. The CpG 1018^®^ is an oligonucleotide adjuvant which consists of a synthetic derivative of CpG-B nucleic acid units connected via a phosphorothioate backbone [34].

### 3.2. Antigenicity of Shigella O-PS-IpaB Conjugate Antigens Formulated with Adjuvants as Measured by Competitive ELISAs

Competitive ELISAs were developed for the four *Shigella* O-PS-IpaB conjugates (*S. sonnei*, *S. f. 3a*, *S. f. 2a,* and *S. f. 6)* to monitor antibody binding (antigenicity) in presence or absence of aluminum salt adjuvants. The key advantage of measuring antibody binding to adsorbed antigens is that it eliminates the need to desorb the antigen from the aluminum salt adjuvant. This competitive ELISA approach thus preserves the structural conformation of the antigen when bound to the adjuvant during analysis, unlike the requirement to desorb the antigen from the adjuvant prior to analysis with sandwich ELISA formats [23,24].

**Figure 2:**
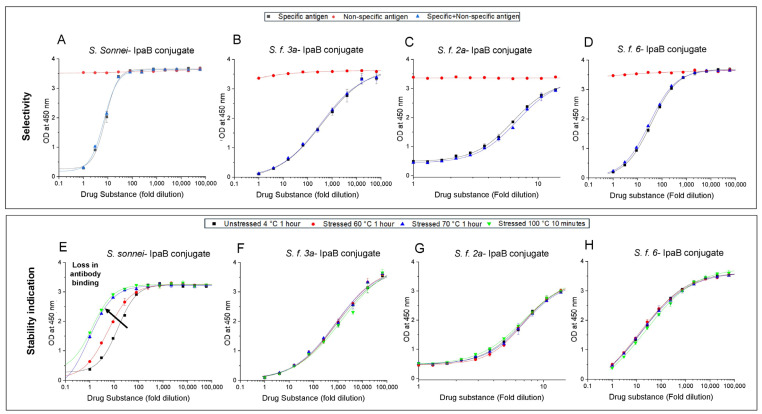
Specificity and stability indication profiles of competitive ELISAs for each of the four unadjuvanted *Shigella* O-PS-IpaB conjugate antigens. (**A**–**D**) Selectivity determinations for each indicated specific conjugate antigen (*S. sonnei*, *S. f. 3a*, *S. f. 2a,* or *S. f. 6)* in presence of non-specific antigens (i.e., all the other antigens excluding the specific antigen) are presented in top panels. (**E**–**H**) Stability-indication using indicated monovalent conjugate antigen by comparing unstressed (stored at 2–8 °C for 1 h) to heat-stressed (60 and 70 °C for 1 h, and 100 °C for 10 min) samples. The drug substance samples contained 2 mM sodium phosphate, 0.15 M NaCl, 0.05% w/v LDAO at pH 7.0. Error bars denote the range from n = 2 (1 vial analyzed at n = 2). Drug substance- conjugate antigen without any adjuvant.

As part of method development, the selectivity of each competitive ELISA was evaluated for each of the four *Shigella* O-PS-IpaB conjugate antigens (in the absence of adjuvant or any environmental stress). Three different samples were compared to demonstrate specificity (i.e., where the “specific antigen” is the antigen to be analyzed and “non-specific antigens” are all the other antigens): (1) a known concentration of the specific antigen, (2) the specific antigen in the presence of non-specific antigens, and (3) non-specific antigens without the specific antigen. As shown in Figure 2A–D, each of the four competitive ELISAs (O-PS-IpaB conjugates of *S. sonnei*, *S. f. 3a, S. f. 2a,* and *S. f. 6*, respectively) was specific for the target antigen only. It was also observed that the assay dilution series differed among the four antigens, a result likely demonstrating varying binding affinities for the four different antigen-specific antibody reagents.

Next, the stability-indicating nature of the four competitive ELISAs was determined in unadjuvanted monovalent antigen formulations (i.e., O-PS-IpaB conjugates of *S. sonnei*, *S. f. 3a, S. f. 2a,* and *S. f. 6*) before and after thermal stress (e.g., either 1 h at either 60 or 70 °C, or 10 min at 100 °C). Results demonstrated an increasing loss in antibody binding with increasing heat stress for *S. Sonnei* O-PS-IpaB conjugate (Figure 2E), while the other three conjugate antigens did not show stability indication (Figure 2F–H). In summary, out of the four conjugates examined, only the competitive ELISA for the *S. Sonnei* O-PS-IpaB antigen displayed stability indication.

**Figure 3:**
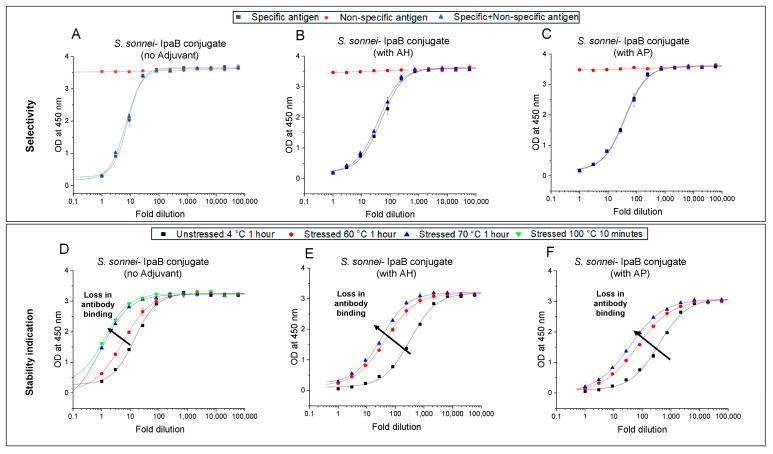
Specificity and stability indication profiles of competitive ELISA for *S. sonnei O-PS-IpaB* conjugate antigen in presence and absence of aluminum salt adjuvants (AH, AP). Selectivity for specific antigen (*S. sonnei* conjugate) with non-specific antigens (trivalent formulation with conjugates of *S. f. 2a, S. f. 3a, S. f. 6*) was determined for (**A**) unadjuvanted antigen, or in the presence of aluminum salt adjuvants (**B**) AH, and (**C**) AP. The stability indicating properties were evaluated for unstressed (stored at 2–8 °C for 1 h) samples vs. (**D**) unadjuvanted antigen after heat stress (60 and 70 °C 1 h, and 100 °C for 10 min), and vs. (**E**,**F**) AH- and AP-adjuvanted antigen samples, respectively, after heat stress (60 and 70 °C 1 h). The conjugate antigen samples contained 2 mM sodium phosphate, 0.15 M NaCl, 0.05% w/v LDAO at pH 7.0 (for panels **A**,**B**,**D**,**E**) and pH 5.8 (panels **C**,**F**). Error bars denote the range from n = 2 (1 vial analyzed at n = 2). AH—Alhydrogel^®^, AP—Adju-phos^®^.

Finally, we evaluated the selectivity and stability indication of the competitive ELISA for the *S. Sonnei* O-PS-IpaB antigen in the presence of aluminum salt adjuvants using the same approach described above. The results confirmed no differences in selectivity of unstressed *S. sonnei* conjugate antigen in absence (Figure 3A) or presence of aluminum salt adjuvants, i.e., AH (Figure 3B) and AP (Figure 3C). For stability indication testing, measurable losses in antibody binding with increasing thermal stress (one hour at 60° and 70 °C) were noted for the *S. sonnei* conjugate antigen compared to the control (one hour at 4 °C), and no additional losses were observed under extreme conditions (100 °C for 10 min) (Figure 3D). Based on these results, the effect of the presence of aluminum adjuvants, i.e., AH (Figure 3E) and AP (Figure 3F) was evaluated (one hour at 60° and 70 °C), and stability indication of the aluminum salt adjuvanted *S. sonnei* conjugate antigen was also demonstrated under thermal-stress conditions.

### 3.3. Antigen–Adjuvant Interactions at Different pH Values

The m-BCA assay was utilized to measure total protein concentration of *Shigella* conjugate antigens in solution to determine the degree of adsorption of the antigens to aluminum salt adjuvants (AH, AP). Monovalent and quadrivalent formulations of the four *Shigella* antigens (O-PS-IpaB conjugates with *S. f. 2a*, *S. f. 3a*, *S. f. 6,* and *S. sonnei*) were prepared in a buffer at either pH 7.0 or pH 5.8 (2 mM sodium phosphate, 0.15 M NaCl, 0.05% w/v LDAO) in the presence of AH or AP (see Section 2.2.1).

**Figure 4:**
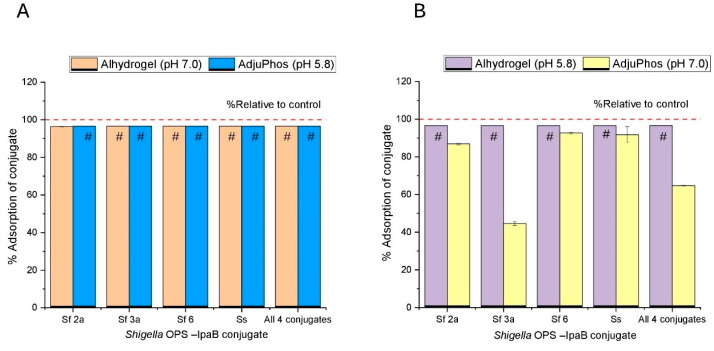
Percent binding of four different *Shigella-IpaB polysaccharide–protein* conjugate antigens to two different aluminum salt adjuvants (AH and AP). Percent adsorption of indicated monovalent conjugate antigens and for the quadrivalent formulation to the indicated aluminum salt adjuvant as measured by the micro-BCA assay (see Section 2.2.3). (**A**) Percent antigen binding under preferred pH conditions (AH at pH 7.0 and AP at pH 5.8), and (**B**) percent antigen binding under non-preferred pH conditions (AH at pH 5.8 and AP at pH 7.0); see text. Formulations contained 2 mM sodium phosphate, 0.15 M NaCl, 0.05% w/v LDAO at pH 7.0 or 5.8. “#” indicates value is equal to or greater than 97% (based on the estimated LOQ of the assay). Error bars denote the range from n = 2 (1 vial analyzed at n = 2). AH—Alhydrogel^®^ and AP—Adju-phos^®^.

Essentially complete antigen adsorption was observed with AH at pH 7.0 and AP at pH 5.8, respectively, for all the monovalent and quadrivalent conjugate antigen formulations (Figure 4A). Similarly, antigen adsorption with AH at pH 5.8 showed essentially complete adsorption of all monovalent and quadrivalent conjugate antigen formulations (Figure 4B). In contrast, binding to AP at pH 7.0 was overall lower for the monovalent and quadrivalent conjugate antigen formulations (Figure 4B). For example, the monovalent formulations of the *S. f. 2a*, *S. f. 6*, and *S. sonnei* conjugate antigens each showed relatively higher adsorption (>80%) compared to either the monovalent *S. f.3a* conjugate antigen (~40%) or the quadrivalent mixture of all four glycoconjugate antigens (~60%).

In summary, the preferred pH conditions were identified for binding of the glycoconjugate antigens to aluminum salt adjuvants, i.e., AH at pH 7.0 (all conjugate antigens bound to AH under physiological conditions) and AP at pH 5.8 (all conjugate antigens bound to AP). These two pH/adjuvant conditions were selected for additional antigen–adjuvant interaction studies using the monovalent *S. sonnei* O-PS-IpaB conjugate antigen as described in the next section.

### 3.4. Conjugate Antigen and CpG Adjuvant Interaction Studies with Aluminum Salt Adjuvants at Selected pH Conditions

We next examined the interactions of the aluminum salt adjuvants with both the glycoconjugate antigen and the CpG adjuvant by evaluating the *S. sonnei* O-PS-IpaB conjugate antigen with the two aluminum salt adjuvants (AH at pH 7.0 and AP at pH 5.8), both in the presence and absence of the CpG oligonucleotide adjuvant. In addition, we utilized four formulation buffers containing either 2 or 200 mM sodium phosphate buffer in 0.15 M NaCl, 0.05% w/v LDAO, at either pH 7.0 or 5.8. We first estimated the isoelectric point (pI) value of the *S. sonnei* O-PS-IpaB conjugate antigen from binding studies to cation exchange spin columns at different solution pHs (see Section 2.2.8). As shown in Supplementary Figure S1, the conjugate antigen is bound to the column from pH 4.0 to pH 6.0 and starts eluting at pH 6.5 through subsequent pH elutions. This result shows that the pI value of the *S. sonnei* O-PS-IpaB conjugate antigen is in the range of pH 6.5–7.0.

**Figure 5:**
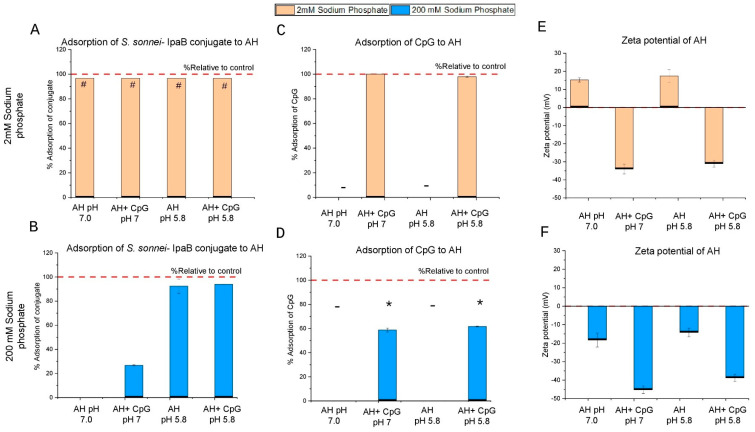
Binding interactions of aluminum hydroxide (AH) adjuvant with *S. sonnei O-PS-IpaB* conjugate antigen and the CpG oligonucleotide adjuvant. (**A**,**B**) Percent adsorption of conjugate antigen to AH at pH 7.0 and 5.8 in 2 and 200 mM phosphate buffers, respectively, as measured using micro-BCA assay. “#” indicates value is equal to or greater than 97% (based on the estimated LOQ of the assay). (**C**,**D**) Percent adsorption of CpG to AH at pH 7.0 and 5.8 in 2 and 200 mM phosphate buffers, respectively, as measured using UV at 260 nm, where “-” indicates formulations without CpG and “*” indicates formulations where CpG has precipitated (see text). (**E**,**F**) Zeta potential values of AH at pH 7.0 and 5.8 in 2 and 200 mM phosphate buffers, respectively, as measured by electrophoretic light scattering (ZetaPals). Formulations contained either 2 or 200 mM sodium phosphate buffer in 0.15 M NaCl, 0.05% w/v LDAO, at either pH 7.0 or 5.8 as indicated. Error bars in panels (**A**,**D**) denote the SD from n = 4 (two vials analyzed at n = 2), panels (**B**,**E**) denote range from n = 2 (1 vial analyzed at n = 2), and panels (**C**,**F**) denote range from n = 2 (one vial analyzed at n = 2). AH—Alhydrogel^®^ and CpG—CpG 1018^®^.

For the aluminum hydroxide (AH) formulations, the *S. sonnei* O-PS-IpaB conjugate antigen was completely adsorbed to AH at both pH 7.0 and 5.8 in the presence and absence of CpG in 2 mM phosphate buffer (Figure 5A). At a higher phosphate (200 mM) concentration, the same conjugate antigen was completely desorbed at pH 7.0, but at pH 5.8 remained ~90% adsorbed to AH (Figure 5B). For the adsorption of CpG to AH, complete binding was observed at 2 mM phosphate both at pH 7.0 and pH 5.8 (Figure 5C) but was reduced to ~60% at 200 mM phosphate, pH 7.0 and pH 5.8 (Figure 5D). To better understand the binding of the conjugate antigen to AH (see Section 4.2), we measured the surface charge of the AH adjuvant in the same formulation buffers. At pH 7.0 and pH 5.8, zeta potential value of AH in 2 mM phosphate was positive (~+15 mV) but in the presence of CpG it changed to negative (~−30 mV) (Figure 5E). At 200 mM phosphate concentration, the zeta potential values for all AH formulations were negative; ~−15 mV in the absence of CpG and ~−40mV in the presence of CpG (Figure 5F).

**Figure 6:**
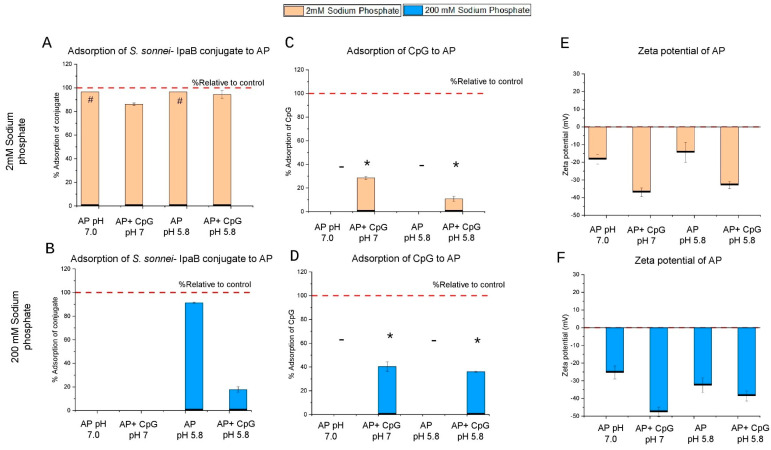
Binding interactions of aluminum phosphate (AP) adjuvant with *S. sonnei O-PS-IpaB* conjugate antigen and the CpG oligonucleotide adjuvant. (**A**,**B**) Percent adsorption of conjugate antigen to AP at pH 7.0 and 5.8 in 2 and 200 mM phosphate buffers, respectively, as measured using micro-BCA assay. “#” indicates value is equal to or greater than 97% (based on the estimated LOQ of the assay). (**C**,**D**) Percent adsorption of CpG to AP at pH 7.0 and 5.8 in 2 and 200 mM phosphate buffers, respectively, as measured using UV at 260 nm, where “-” indicates formulations without CpG and “*” indicates formulations where CpG has precipitated (see text). (**E**,**F**) Zeta potential values of AP at pH 7.0 and 5.8 in 2 and 200 mM phosphate buffers, respectively, as measured by electrophoretic light scattering (ZetaPals). Formulations contained either 2 or 200 mM sodium phosphate buffer in 0.15 M NaCl, 0.05% w/v LDAO, at either pH 7.0 or 5.8 as indicated. Error bars in panels (**A**,**D**) denote the SD from n = 4 (two vials analyzed at n = 2), panels (**B**,**E**) denote range from n = 2 (1 vial analyzed at n = 2), and panels (**C**,**F**) denote range from n = 2 (one vial analyzed at n = 2). AP—Adju-phos^®^ and CpG—CpG 1018^®^.

For aluminum phosphate (AP) formulations, the *S. sonnei* O-PS-IpaB conjugate antigen was essentially all bound to AP in 2 mM phosphate formulations at both pH 7.0 and 5.8, except in the presence of CpG at pH 7.0 where it was ~85% bound (Figure 6A). At a higher phosphate (200 mM) concentration, the same conjugate antigen was completely desorbed at pH 7.0, but at pH 5.8 remained ~90% adsorbed to AP without CpG and was ~20% bound in the presence of CpG (Figure 6B). For the adsorption of CpG adjuvant to AP, at 2 mM phosphate, only partial CpG binding to AP was observed, ~30% at pH 7.0 and ~10% at pH 5.8 (Figure 6C). At 200 mM phosphate, ~40% CpG was bound to AP (Figure 6D). To better understand the observed antigen–adjuvant interactions (see Section 4.2), we measured the surface charge of the AP adjuvant under the same conditions. At pH 7.0 and pH 5.8, zeta potential values of AP in 2 mM phosphate were negative (~−15mV and ~−35 mV) in the absence and presence of CpG (Figure 6E). At 200 mM phosphate concentration, the zeta potential values for all AP formulations were also negative; ~−25 mV in the absence of CpG and ~−40mV in the presence of CpG (Figure 6F). During these experiments, varying levels of CpG were measured in formulations containing AP, a result likely related to the lack of binding of CpG to AP and the presence of the LDAO detergent in the buffer leading to precipitation (as described in more detail below).

### 3.5. Langmuir Binding Isotherm Analysis of O-PS-IpaB Conjugate Antigens with Aluminum Salt Adjuvants

**Figure 7:**
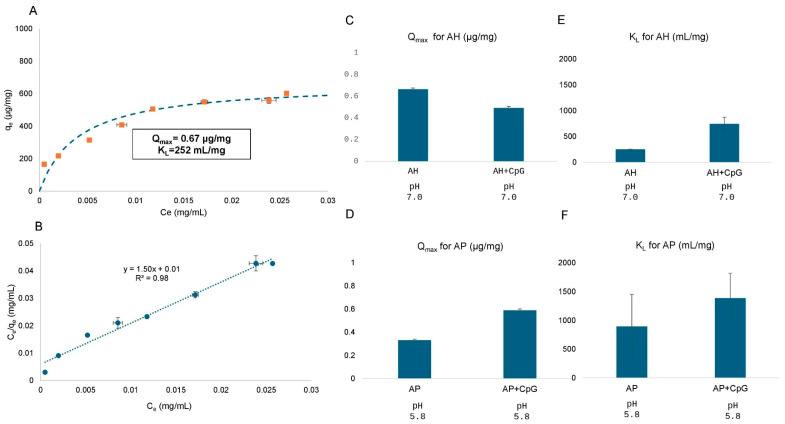
Langmuir binding isotherm analysis of binding of *S. sonnei* O-PS-IpaB conjugate antigen with two aluminum salt adjuvants (AH, AP). (**A**,**B**) Langmuir isotherm and linearized Langmuir isotherm analysis, respectively, conjugate antigen binding data with AH. See Supplementary Figure S2 for binding isotherm plots for the other three formulations (AH+CpG, AP, AP+CpG). (**C**,**D**) Maximum binding capacity values (*Q_max_*) conjugate antigen to AH and AP, respectively, in the presence and absence of CpG adjuvant. (**E**,**F**) Strength of interaction values (*K_L_*) of conjugate antigen to AH and AP, respectively, in the presence and absence of CpG adjuvant. Formulations contained 2 mM sodium phosphate, 0.15 M NaCl, 0.05% w/v LDAO at pH 7.0 for AH and pH 5.8 for AP. Error bars in panels (**A**,**B**) denote the range from n = 2 (1 vial analyzed at n = 2), panels (**C**,**D**) denote SD from n = 4 (2 vials analyzed at n = 2), and panels (**E**,**F**) denote range or SD from n = 2–4 (1–2 vials analyzed at n = 2). AH—Alhydrogel^®^, AP—Adju-phos^®^, and CpG—CpG 1018^®^.

To further evaluate the binding interactions of the *S. sonnei* O-PS-IpaB conjugate antigen to AH and AP adjuvants in the presence and absence of CpG, we performed Langmuir isotherm binding experiments (see Section 2.2.6). Representative data fits for Langmuir isotherm and linearized Langmuir isotherm for conjugate antigen with AH adjuvant are shown in Figure 7A and Figure 7B, respectively. Similar Langmuir isotherm binding data sets for the same conjugate antigen in the three other formulations (AH+CpG, AP, and AP+CpG) are shown in Supplementary Figure S2.

The linearized Langmuir isotherm model, assuming monolayer coverage, was then used to calculate the binding capacity (*Q_max_*) and strength of interaction (*K_L_*) of the conjugate antigen under various formulation conditions. For these experiments, AH (pH 7.0) and AP (pH 5.8) formulations were prepared in the lower phosphate buffer (2 mM sodium phosphate, 0.15 M NaCl, 0.05% w/v LDAO), in the presence or absence of CpG adjuvant. For the AH formulations (Figure 7C,E), the *S. sonnei* O-PS-IpaB conjugate antigen showed small trends (given experimental variability) toward reduced binding capacity values (*Q_max_*) and increased strength of interaction (*K_L_*) values in the presence of CpG. For the AP formulations (Figure 7D), an opposite modest trend was observed with the *Q_max_* value of the *S. sonnei* O-PS-IpaB conjugate antigen’s binding to AP increasing in the presence of CpG. The strength of interaction (*K_L_*) values between the conjugate antigen and AP, however, were comparable in the presence and absence of CpG.

### 3.6. Evaluating CpG Binding to Aluminum Salt Adjuvants and Loss of CpG Material Under Certain Solution Conditions

As described above, the negatively charged CpG oligonucleotide adjuvant does not bind to aluminum salt adjuvants with a net negative surface charge by zeta potential measurements (i.e., AH in the presence of high concentration of phosphate buffer and AP at all phosphate buffer concentrations). At the same time, under these aluminum salt formulation conditions in which CpG is unbound and thus in solution, a lack of mass balance of CpG across the stability experiments was noted indicating loss of material.

To better understand these observations, we isolated the components of the formulation buffer used in previous experiments and prepared the following solutions at 0.6 mg/mL of CpG in the absence of any aluminum salt adjuvant (see Section 2.2.1): (1) 0.15 M NaCl, 0.05% w/v LDAO at pH 7.0 and 5.8 (no phosphate buffer), (2) 2 mM sodium phosphate 0.05% w/v LDAO at pH 7.0 and 5.8 (no sodium chloride), and (3) 2 mM sodium phosphate, 0.15 M NaCl at pH 7.0 and pH 5.8 (no LDAO). We also made comparisons to the buffer used in the stock solution of CpG (20mM Tris 0.15M NaCl, pH 7.5) as a control. We observed that Solution (3) did not show any precipitation, while Solutions (1) and (2) did display precipitation, a result indicating the detergent LDAO causes CpG to precipitate.

**Figure 8:**
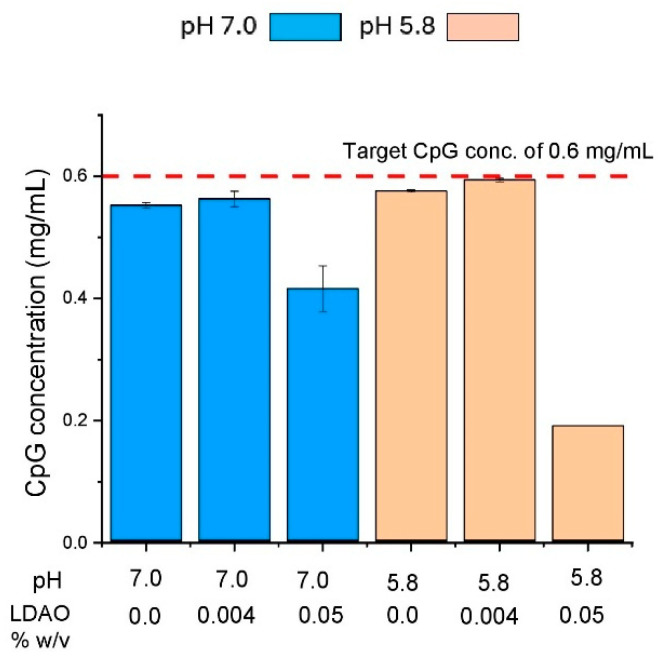
Mitigating the precipitation of the CpG oligonucleotide adjuvant in the formulation buffer by optimizing solution pH and LDAO detergent concentration. The CpG adjuvant concentrations in different formulations were measured by Absorbance values at 260 nm after rotating at room temperature for two hours and storage at 2–8 °C overnight. (see Section 2.2.4). Formulations contained 0.6 mg/mL CpG in 2 mM sodium phosphate buffer, 0.15 M NaCl with varying LDAO concentrations (0, 0.004, and 0.05% w/v) at two different pHs (7.0 and 5.8). The formulations did not contain either antigen or aluminum salt adjuvants. Error bars denote SD from n = 3 (one vial analyzed at n = 3). CpG—CpG 1018^®^.

Based on these results, we then again prepared CpG-alone formulations (with no aluminum salt adjuvant) in a 2 mM sodium phosphate, 0.15 M NaCl buffer at pH 7.0 and 5.8 at three different concentrations of the detergent LDAO: (1) no LDAO, (2) 0.004% w/v LDAO (carryover LDAO levels from the bulk antigens when preparing a quadrivalent conjugate antigen formulation), and (3) 0.05% w/v LDAO (the level present in the bulk antigen solutions). The CpG was mixed with these solutions (2 h by rotation at room temperature and stored at 2–8 °C overnight), and the amount of CpG present was determined. As shown in Figure 8, at both pH values, the CpG recovery (by comparison to the no LDAO control buffer) was lower at 0.05% w/v LDAO, but full recovery was observed in 0.004% w/v LDAO-containing buffer. These results indicate that at higher levels of the LDAO detergent, there was a lack of mass balance for the CpG adjuvant, most likely due to precipitation of the complex. In fact, we could visually observe precipitates in the CpG solutions at pH 5.8 but not at pH 7.0 (with LDAO at 0.05% w/v). Since similar experiments could not be performed in the presence of AP adjuvant (the colloidal AP adjuvant interferes with ability to visually observe CpG precipitants), we utilized these CpG solution formulation results to design vaccine formulations containing glycoconjugate, CpG, and aluminum salt adjuvant, as described below.

### 3.7. Preparation of 12 Different Adjuvanted Formulations and Effects of Increasing Temperature Exposure on Antigenicity of S. sonnei O-PS-IpaB Conjugate Antigen

Next, after identifying the most likely cause of poor CpG recovery as precipitation of CpG-LDAO complexes formed at a higher LDAO concentration, conjugate antigen formulations at 0.004% w/v LDAO concentration (estimated level found in quadrivalent conjugate antigen formulations) and 0.001% w/v LDAO (estimated levels for monovalent conjugate antigen formulations) were prepared for further studies. These formulations contained either AP and AH adjuvants at two different pH values in the presence and absence of CpG.

**Figure 9:**
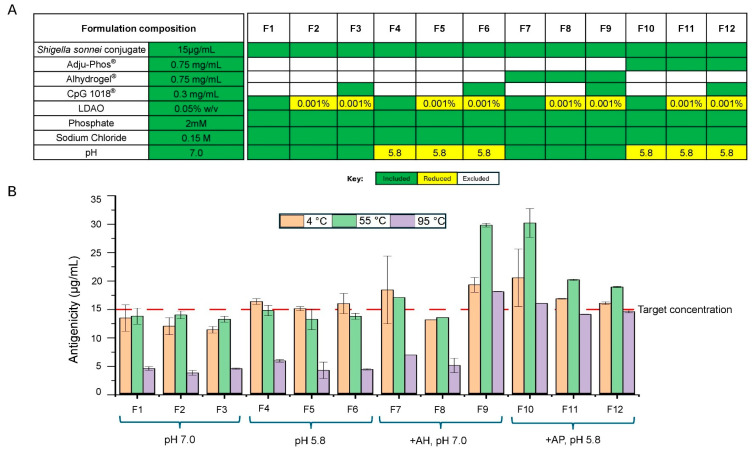
Summary of 12 different formulations of monovalent *S. sonnei* O-PS-IpaB conjugate antigen (with zero, one, or two adjuvants). (**A**) Design and composition of the 12 formulations evaluated in thermal stress and storage stability studies. See Section 2.2.9 for details of the preparation of each formulation. (**B**) Antigenicity (µg/mL) of the 12 formulations that are heat-stressed for an hour at temperatures at 55 °C and 95 °C with comparisons shown with the control stored at 4 °C for three days. F9, F11, and F12 results were obtained separately due to experimental errors during the head-to-head comparisons. Error bars denote range or SD from n = 2–8 (1–4 vials analyzed at n = 2).

These twelve formulations were designed to evaluate the effects of aluminum salt adjuvants, CpG adjuvants, solution pH, and carryover LDAO concentrations on the antigenicity and stability of the monovalent *S. sonnei* O-PS-IpaB conjugate antigen. As shown in Figure 9A, F1–F6 are solution formulations without aluminum salt adjuvant, and F7–F12 are aluminum salt adjuvanted formulations containing either AH at pH 7.0 or AP at pH 5.8. The 12 formulations (F1–F12) varied in terms of CpG and LDAO levels, but all were weakly buffered and isotonic (i.e., 2 mM phosphate and 0.15 M NaCl).

These 12 different formulations of *S. Sonnei* O-PS-IpaB conjugate antigen were evaluated for thermal degradation mechanisms (this section) and storage stability studies (next section). For the former, the twelve formulations were prepared at 2–8 °C and then exposed to temperatures ranging from 55 to 95 °C, with an increase of 5 °C consecutively for one hour at each temperature. Antigenicity values were then determined for all samples and two different thermal effects were noted. First, in the lower to elevated temperature ranges (i.e., at 2 to 8 °C and at 55 °C), samples displayed either no change in antigenicity, or in some cases, an increase in antigenicity values at elevated temperatures for a few of the samples, as shown in Figure 9B (also see Section 4.3). Second, in the higher temperature ranges (i.e., at 95 °C), samples exhibited a decrease in antigenicity values with varying rates of degradation depending on the formulation and adjuvant, as displayed in Figure 9B.

**Figure 10:**
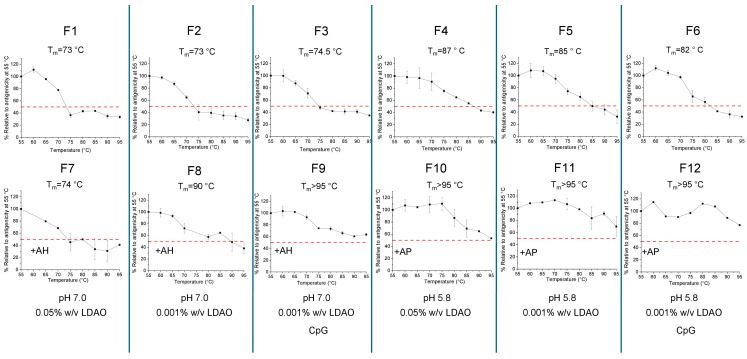
Thermal degradation profiles of monovalent *S. sonnei* O-PS-IpaB conjugate antigen in 12 different formulations as measured by competitive ELISA. Antigen–antibody binding results for conjugate antigen in 12 different formulations (F1–F12; see Figure 9A for composition) during thermal stress (incubation for one hour at indicated temperature range). Stability profiles are plotted as relative antigenicity values normalized to antigenicity values at 55 °C (see Figure 9B) vs. incubation temperature. (Panels F1–F6) Conjugate antigen formulations containing formulation buffer (0.05% LDAO), formulation buffer with lower LDAO concentration (0.001%), or formulation buffer with both lower LDAO concentration and addition of CpG adjuvant. (Panels F7–F9) Conjugate antigen + AH adjuvant formulations containing formulation buffer (0.05% LDAO), formulation buffer with lower LDAO concentration (0.001%), or formulation buffer with both lower LDAO concentration and addition of CpG adjuvant. (Panels F10–F12) Conjugate antigen + AP adjuvant formulations containing formulation buffer (0.05% LDAO), formulation buffer with lower LDAO concentration (0.001%), or formulation buffer with both lower LDAO concentration and addition of CpG adjuvant. See Section 2.2.9 for preparation of formulations. F9, F11, and F12 results were obtained separately due to experimental errors during the head-to-head comparisons. Error bars denote range or SD from n = 2–8 (1–4 vials analyzed at n = 2).

To better understand the effects of each formulation variable on the thermal degradation profile of the antigenicity, we plotted the relative loss in antigenicity for every 5 °C temperature increment in the higher temperature ranges between 55 and 95 °C, with all values normalized to 100% of the 55 °C value (Figure 10), and determined Tm values, the temperature where 50% loss of the relative antigenicity values, were observed. We first examined the effect of aluminum salt adjuvants and solution pH on the stability of the *S. Sonnei* O-PS-IpaB conjugate antigen. The antigen alone in three different solution conditions at pH 7.0 (F1, F2, F3 in Figure 10) displayed Tm values of ~73–75 °C. When the AH-adsorbed antigen was incubated in same three pH 7.0. formulations, the observed Tm values were similar (F7) or higher (~90 or >95 °C) (F8, F9 in Figure 10). In contrast, when formulated in the same three solution conditions at pH 5.8 (F4, F5, F6 in Figure 10), the unadjuvanted antigen displayed Tm values of ~82–87 °C. This result demonstrated the antigen is more stable at pH 5.8 (vs. pH 7.0) at elevated temperatures. Finally, when the AP-adsorbed antigen was incubated in the same three pH 5.8 formulations, the observed Tm values were much higher (>95 °C) (F10, F11, F12 in Figure 10). In summary, the best thermal stability profile of the conjugate antigen was noted when absorbed to AP adjuvant in formulations at pH 5.8.

The effect of LDAO concentration and the CpG adjuvant was also evaluated in the thermal stability experiments presented in Figure 10. For unadjuvanted formulations, no notable effects were seen for either additive at either pH 7.0 (F1 vs. F2 vs. F3) or pH 5.8 (F4 vs. F5 vs. F6). In AH adjuvanted formulations at pH 7.0, lower LDAO concentrations resulted in improved thermal stability with a shift in Tm values from 74 °C in F7 (0.05% LDAO) to ~90 °C in F8 (0.001% LDAO). In AP adjuvanted formulations at pH 5.8, however, no effect of LDAO was noted with similar Tm values observed at both LDAO concentrations. Finally, the presence of CpG showed a slightly increased Tm in AH adjuvanted formulations from 90 °C (no CpG) to greater than 95 °C (with CpG). In the other formulations, CpG adjuvant had minimal effect on the thermal stability profile of the antigen.

### 3.8. Short-Term Storage Stability Study at Different Temperatures

**Figure 11:**
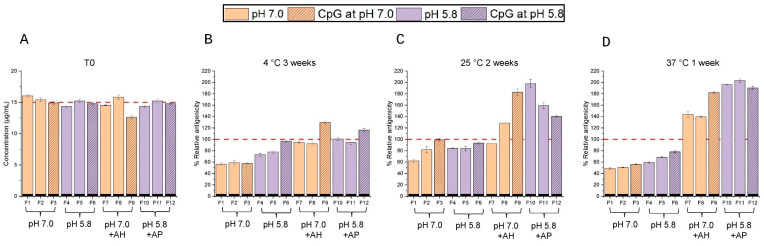
Short-term storage stability study at different temperatures of monovalent *S. sonnei* O-PS-IpaB conjugate antigen in 12 different formulations as measured by competitive ELISA. (**A**) Antigenicity values (µg/mL) of the conjugate antigen in 12 different formulations at time zero (see Figure 9A for composition, F1–F12). (**B**–**D**) Relative stability profiles of antigen–antibody binding results (percent of T = 0 values) for the same conjugate formulations after incubation at (**B**) 2–8 °C for 3 weeks, (**C**) 25 °C for 2 weeks, and (**D**) 37 °C for 1 week. See Section 2.2.9 for preparation of formulations. Error bars denote SD from n = 6 (3 vials each analyzed at n = 2).

The same twelve formulations described above of the *S. sonnei* O-PS-IpaB conjugate antigen (Figure 9A) were also evaluated in a short-term real-time and accelerated stability study. Stability samples were stored in pharmaceutical vials/stoppers and antigenicity values were measured using competitive ELISA (see Section 2.2.2). The 12 formulations were incubated at 2–8 °C (3 weeks), 25 °C (2 weeks), and 37 °C (1 week) and the stability profiles were plotted with respect to normalized time zero results (T0). At T0 (Figure 11A), all formulations displayed antigenicity values of 15 ± 3 µg/mL, which were in good agreement with the target value of 15 µg/mL. The target levels of CpG in solution formulations F3, F6, and F12, along with the binding of CpG to the AH adjuvant, were confirmed, as shown in Supplementary Figure S3.

After incubation at 2–8 °C for 3 weeks (Figure 11B), the aluminum salt adjuvanted formulations (F7-12) were stable (~100–120% relative antigenicity), but instability was seen in most of the solution formulations. For the solution formulations, F1-F3 exhibited ~60% relative antigenicity remaining while F4 and F5 showed ~75% relative antigenicity remaining. Interestingly, formulation F6 displayed better stability with ~90% relative antigenicity remaining. This formulation contained CpG, lower LDAO levels, and was at pH 5.8.

During accelerated storage at 25 °C for 2 weeks (Figure 11C) and 37 °C for 1 week (Figure 11D), similar trends were noted for improved stability of the conjugate antigen when (1) formulated with aluminum salt adjuvants compared to solution and (2) for aluminum salt adjuvanted samples when bound to AH at pH 7.0 and AP at pH 5.8. Moreover, the aluminum salt adjuvanted conjugate antigen samples stored at elevated temperatures for 1–2 weeks had relative antigenicity values higher than T0, a trend overall more prominent in AP than AH adsorbed antigen samples (see Section 4.3).

## 4. Discussion

### 4.1. Antigen Structural Complexity, Analytical Challenges, and Suggested Future Work

In general, the structural complexity of a glycoconjugate vaccine candidate arises from multiple factors, including the polysaccharide antigen(s), the carrier protein, the conjugation reaction linking the two components, the drug product’s multivalent composition, and formulation components, including excipients and adjuvants. Such structural and formulation complexities present many analytical challenges to characterize glycoconjugate vaccine candidates for clinical development [35]. The conjugate vaccine candidate evaluated in this work consisted of four different *Shigella* O-PSs linked to IpaB carrier protein. Chemical conjugation methods, like reductive amination and cyanylation using CDAP, result in conjugates with a broad size range and ill-defined physicochemical properties due to the presence of multiple lysine residues in a protein that undergo chemical conjugation [32]. Further, batch-to-batch variability in antigen preparation as well as the degree of conjugation after coupling and long-time storage also contributes to vaccine heterogeneity [36].

In this work, the complexity of the conjugate antigen included the nature of the carrier protein. The IpaB protein is highly hydrophobic and requires addition of the detergent LDAO for isolation from its chaperone protein, IpgC, and storage in solution without aggregation [37]. For this reason, the bulk solutions of the conjugate antigens contained 0.05% w/v LDAO (which is at a higher concentration than its critical micelle concentration of 0.023%) [38]. The presence of zwitterionic detergent micelles skews results from common analytical techniques for measurement of the conjugate antigen size including DLS and SEC-HPLC. Further, the large and heterogeneous structure of conjugate antigens limited the ability to perform isoelectric point assessments using capillary isoelectric focusing.

Additionally, the inclusion of adjuvants (i.e., AH, AP, and CpG-1018) leads to further structural complexity of a vaccine candidate. Adjuvants are typically required to lower the dose of conjugate antigens (in µg ranges) while enhancing the protective immunity for subunit vaccines [39,40,41]. Concomitantly, however, aluminum salt adjuvants can potentially destabilize antigens by inducing aggregation or structural alterations [42,43], but such destabilizing effects are both antigen specific and dependent on formulation conditions. This possibility of adjuvant-induced destabilization of the glycoconjugate antigen was a major motivation to perform the studies described in this work. As a final point, the particulate nature of aluminum salt adjuvants, along with heterogeneity of antigen–adjuvant interactions (i.e., bound vs. unbound antigen to aluminum salts), introduces additional analytical challenges when characterizing formulated preparations of the glycoconjugate antigens.

An estimated 70% of the overall manufacturing time for producing a combination vaccine is attributed to QC assays [44]. Since in vivo potency assays of *Shigella* conjugates (e.g., immunogenicity readouts in rabbits) are more labor-intensive, costly, and prone to variability of biological readouts, we focused in this work on in vitro assays like ELISA (binding of conjugate antigen to antigen-specific antibodies). Additionally, there has been increasing interest in reducing, replacing, and refining the use of animals through alternative analytical methods that closely correlate with animal potency assay results (known as the 3Rs principles) [45]. Notable examples of animal-based potency assays replaced by immunochemical binding methods (i.e., in vitro potency assays) for human vaccines include those for hepatitis A and B, inactivated poliovirus, and human papillomavirus [45].

To facilitate analysis for formulation development work described herein, we developed competitive ELISAs to assess the antigenicity of *Shigella* conjugates when bound or unbound to aluminum salt adjuvants. Competitive ELISA bypasses the requirement to release bound antigen (i.e., desorb the conjugate antigen from aluminum salt adjuvant surface or dissolve the aluminum salt adjuvant), thus allowing for the analysis of an intact antigen in its immunogenic form on the aluminum salt adjuvant. As described in the Section 3, although all four competitive ELISAs were developed using selected antigen-specific capture antibodies that demonstrated selectivity, only *S. sonnei* assay was stability indicating. Therefore, our in vitro stability studies focused on *S. sonnei* O-PS-IpaB conjugate to identify compatible formulations with aluminum salt and CpG adjuvants. The capture antibodies specifically targeted the polysaccharide portion of the conjugates which is known to be generally heat-stable [46] (in contrast to carrier protein). The observed loss of antibody binding in stressed *S. sonnei* samples may potentially be attributed to steric hinderance caused by structurally altered IpaB protein interfering with the antibody binding site on the polysaccharide surface.

In the future, it will be helpful to identify improved ELISA antibodies to evaluate the stability of the remaining three *Shigella* O-PS-IpaB conjugates (i.e., *S. f. 3a, 2a, and 6*), as well as implement a more comprehensive ELISA method development approach using neutralizing monoclonal antibodies to better correlate in vitro binding and in vivo immunogenicity results. It is important to note that the ELISA results in this work are not necessarily linked to in vivo immunogenicity outcomes from animal studies, which will require future investigations.

### 4.2. S. sonnei O-PS-IpaB Conjugate Antigen and CpG Adjuvant Interaction Studies with Aluminum Salt Adjuvants and Suggested Future Work

Binding interactions between vaccine antigens (and the CpG adjuvant) with aluminum salt adjuvants may affect both in vivo immunopotentiation as well as in vitro storage stability profiles [42,47,48,49]. The extent of binding of the *Shigella* O-PS-IpaB conjugate antigens as well as the CpG adjuvant to the surface of the colloidal suspensions of aluminum salt adjuvants (i.e., AH or AP) depends on the nature of their molecular interactions, including electrostatic, hydrophobic, Van der Waals, ligand exchange, etc. For electrostatic interactions, AH has a point of zero charge (PZC) at pH 11 whereas AP has a PZC at pH 4.6–5.6, depending on the phosphate and hydroxyl stoichiometry [47]. Therefore, at pH 7.0, the surface of the AH and AP adjuvants is positively and negatively charged, respectively.

In this work, we determined the pI value of *Shigella* O-PS-IpaB conjugate antigen to be in the range of pH 6.5–7.0 (see Supplementary Figure S1). In addition, there is a net neutral charge for the zwitterionic detergent LDAO at pH 7.0 and net negative charge for the CpG adjuvant under these conditions. Taking these experimental results into consideration, we evaluated the possible binding mechanisms of each component to the aluminum salt adjuvants under different solution and formulation conditions. A summary of the experimental results and possible binding mechanisms are provided in Table 1 for AH and Table 2 for AP and discussed below.

#### 4.2.1. Effect of pH on CpG Adjuvant and Glycoconjugate Antigen Interactions with Aluminum Hydroxide (AH) Adjuvant

In the case of AH adjuvant at 2 mM phosphate (Table 1), the AH surface remains overall positively charged at both pH 5.8 and 7.0 (due to the low phosphate concentration compared to 200 mM phosphate formulation; see below). When negatively charged CpG adjuvant is added, the AH surface charge becomes negative and complete binding of the CpG to AH is observed. Another mechanism of binding of CpG to AH could be ligand exchange (phosphorothioate in CpG and hydroxyl in AH) [50]. Interestingly, the *S. sonnei* O-PS-IpaB conjugate antigen binds under all four conditions, yet the antigen has a net negative charge at pH 7.0 and a net positive charge at pH 5.8. These results indicate that, although electrostatic interactions play a role in antigen–AH adjuvant interactions, other molecular interactions likely also contribute under these conditions. In this context, “other molecular interactions” (highlighted in Table 1 for AH and Table 2 for AP) between the glycoconjugate antigen and aluminum salt adjuvants (AH, AP) includes noncovalent forces such as hydrophobic and Van der Waals covalent interactions such as ligand exchange, but also cannot exclude the possibility of distributions of both negatively and positively charged patches on the antigen surface leading to unexpected electrostatic interactions that are not expected based on the overall pI measurements).

#### 4.2.2. Effect of Phosphate Buffer on CpG Adjuvant and Glycoconjugate Antigen Interactions with Aluminum Hydroxide (AH) Adjuvant

For the AH adjuvant at the higher 200 mM phosphate concentration (Table 1), phosphate ions exchange with hydroxyls on the AH surface via ligand exchange to a much greater extent (compared to the 2 mM phosphate formulation; see above) resulting in an alteration of the surface zeta potential values to a net negative charge at both pH 7.0 and 5.8. When negatively charged CpG adjuvant is added, it does not bind the 200 mM phosphate-treated AH and material is lost (presumably due to precipitation since CpG is in solution and can interact with the LDAO detergent). This result contrasts with CpG adsorbed on the positively charged AH in the 2 mM phosphate formulation (i.e., adsorbing CpG to AH prevents precipitation with the LDAO in solution). Under these conditions at 200 mM phosphate buffer, the *S. sonnei* O-PS-IpaB conjugate antigen essentially does not bind to AH at pH 7.0 (both net negative charge) and does bind at pH 5.8 (with conjugate and AP having a net positive and negative charge, respectively), a result consistent with electrostatic forces dominating antigen–adjuvant binding interactions under these conditions.

#### 4.2.3. Effect of pH on CpG Adjuvant and Glycoconjugate Antigen Interactions with Aluminum Phosphate (AP) Adjuvant

For the AP adjuvant at 2 mM phosphate concentration (Table 2), the surface zeta potential value was a net negative charge at both pH 7.0 and 5.8. When CpG adjuvant is added, it does not bind to AP, a result consistent with previous work in our lab that demonstrated CpG does not bind to AP surface due to electrostatic repulsion as both are negatively charged [26,33]. In this case, CpG is partially lost in the experiment, a result likely due to precipitation upon interacting with LDAO in solution. For the glycoconjugate–AP interactions under these conditions, the *S. sonnei* O-PS-IpaB conjugate antigen binds essentially completely to AP under all four conditions. These results indicate that although electrostatic interactions play a role in antigen–AP adjuvant interactions, other molecular interactions also contribute under these conditions.

#### 4.2.4. Effect of Phosphate Buffer on CpG Adjuvant and Glycoconjugate Antigen Interactions with Aluminum Phosphate (AP) Adjuvant

For the AP adjuvant at 200 mM phosphate concentration (Table 2), the surface zeta potential value was a net negative charge at both pH 7.0 and 5.8. When negatively charged CpG adjuvant is added, it does not bind to AP and CpG material is lost, a result likely due to precipitation in the presence of LDAO detergent. Under these conditions at pH 7.0, the *S. sonnei* O-PS-IpaB conjugate antigen is also negatively charged and does not bind to AP. Since the antigen does bind at 2 mM phosphate (Table 2), this result implies the additional phosphate further alters the AP surface which is consistent with the increased negative zeta potential values measured under these conditions. At pH 5.8, the *S. sonnei* O-PS-IpaB conjugate antigen is now positively charged and does bind to the AP adjuvant. Upon addition of CpG, however, the antigen was surprisingly mostly not bound (~80%) to AP adjuvant. We speculate this result may perhaps be due to displacement by CpG-LDAO complexes.

In terms of future work, a better understanding of the role of non-electrostatic forces in antigen–adjuvant binding is needed, including potential roles of (1) the zwitterionic detergent LDAO which becomes more positively charged at lower pH values, (2) potential conformational changes that may occur within the antigen at lower pH values, and (3) pockets of positive and negative charges on the complex antigen under the assumption of a net surface charge that could be responsible for interactions with adjuvants. In this work, the CpG remaining in solution was susceptible to increasing precipitation in the presence of LDAO from pH 7.0 to pH 5.8, likely due to the LDAO detergent becoming more positively charged and interacting with the negatively charged CpG. Since the presence of LDAO is incompatible with unbound CpG, future antigen bulks should be tested at a lower LDAO concentration or without any LDAO. Although removal of LDAO should facilitate the development of stable AP formulations, with CpG, it is possible it may result in destabilization of the glycoconjugate antigen.

### 4.3. Stability Studies with Different Adjuvanted Formulations of S. sonnei O-PS-IpaB Conjugate Antigen and Suggested Future Work

#### 4.3.1. Antigen Stability in Different Aluminum Salt Adjuvanted Formulations

Heat-stress and storage stability studies showed that the *S. sonnei* O-PS-IpaB conjugate antigen was overall more stable when bound to aluminum salt adjuvant than in solution. In addition, the aluminum salt adjuvanted formulations showed consistently higher antigenicity values when incubated at elevated temperatures (i.e., 25 for 2 weeks and 37 °C for 1 week), an observation we have reported previously with other vaccine antigens, hypothetically due to conformational changes within the antigen when interacting with the adjuvant surface that result in more epitopes being exposed [51]. Previous studies with protein antigens have shown that antigens can be structurally altered upon adsorption to the aluminum salt adjuvant surface [52]. During the thermal stress studies, the best stability profile of the conjugate antigen was noted when adsorbed to AP adjuvant in formulations at pH 5.8. These conditions also resulted in the highest antigenicity values in the storage stability studies, a result showing the heat-stress studies may be an early indicator of trends observed during longer-term storage stability studies.

The stability comparisons of the different adjuvanted formulations evaluated in this work were based solely on *S. sonnei* OPS-IpaB antigen. As part of future work, long-term storage stability studies (i.e., 1–2 years) with all four *Shigella* O-PS-IpaB conjugate antigens are suggested for establishing the stability profiles and determining the degradation mechanism(s) of each of the conjugate antigens in different adjuvanted formulations. Previous work has shown the presence of phosphate or acetyl groups to be a causative factor for depolymerization of polysaccharide portion of other glycoconjugate antigens [53,54]. Since *S. sonnei* O-PS lacks phosphate groups and acetyl groups, further investigation is needed to determine if chemical depolymerization is a cause of antigen instability. As mentioned above, another possibility for loss of antibody binding during storage may be attributed to steric hinderance caused by structurally altered IpaB carrier protein interfering with the antibody binding site on the polysaccharide surface.

It has been reported that antibody binding results from ELISAs can be more sensitive to subtle structural changes within antigens during storage compared to animal immunogenicity tests [55]. Future studies should consider combining in vitro potency assays (antigenicity by competitive ELISA) and in vivo potency assays (animal immunogenicity) for a more comprehensive understanding of the stability profiles of the four *Shigella* O-PS-IpaB conjugates in the different adjuvanted formulations.

#### 4.3.2. CpG Adjuvant Stability in Different Aluminum Salt Adjuvanted Formulations and Selection of Candidate Formulations

To incorporate the oligonucleotide CpG adjuvant into the *S. sonnei* O-PS-IpaB conjugate antigen formulations, we evaluated formulations in solution and in the presence of aluminum salt adjuvants. First, in terms of antigen stability, no notable stabilizing or destabilizing effects were noted for CpG across stability studies in any of the formulations. Second, instability of CpG in solution was an issue with precipitation noted in a solution containing 0.05% LDAO, an effect that was minimized when lowering the LDAO concentration to 0.004%. However, when we subsequently evaluated lower LDAO concentrations of 0.001% and 0.004% (w/v) for *S. sonnei* O-PS-IpaB conjugate antigen formulations during storage stability studies, we identified smaller amounts of CpG in formulations in solution (F3 and F6), implying some precipitation of CpG was still occurring at these low LDAO levels (see Supplementary Figure S3). We also noted a lower CpG concentration in the AP-containing formulation (F12) at low LDAO concentrations, presumably due to the same precipitation mechanism. Thus, the development of a stable AP-CpG formulation of the *Shigella* O-PS-IpaB conjugate antigens requires the bulk conjugate antigen solutions to be reoptimized to no longer contain the detergent LDAO. Such a reformulation effort to replace LDAO with an alternative detergent may or may not be successful in preventing aggregation and instability of the bulk conjugate antigen.

In contrast, AH-CpG formulations at pH 7.0 of the *S. sonnei* O-PS-IpaB conjugate antigen looked promising since (1) the CpG was bound on AH surface and thus did not precipitate in the presence of LDAO, and (2) displayed good storage stability and antigenicity during storage for several weeks. This AH-CpG formulation is therefore sufficiently stable to be evaluated in vivo immunogenicity models to assess if the CpG adjuvant can further enhance the immunogenicity of aluminum salt adjuvanted formulations of the quadrivalent *Shigella* O-PS-IpaB conjugate vaccine candidate.

## 5. Conclusions

In this work, we evaluated the stability profiles of adjuvanted formulations of four *Shigella* glycoconjugate antigens, i.e., serotype-specific O-PS purified from four different *Shigella* strains (*S. flexneri*, *2a*, *3a*, and *6*, and *S. sonnei*) and chemically conjugated to the carrier protein IpaB. We focused on aluminum salt (AP, AH) and oligonucleotide (CpG 1018) based adjuvants, since (1) each adjuvant is used in commercial vaccine products and thus should be more readily available at lower costs compared to novel adjuvants and (2) have the potential of together better stimulating the immune system via different mechanisms, potentially providing a synergistic protective effects (i.e., Th1 and Th2 responses induced by CpG and aluminum salt adjuvants, respectively [41,48]). The main goal of this work was to identify stabilizing formulation, solution, and storage conditions (for both the antigens and the adjuvants) to enable future in vivo animal immunogenicity and long-term storage stability studies.

To this end, we developed serotype-specific competitive ELISAs for each of the four *Shigella* OPS-IpaB antigens. We then focused on evaluating antigen compatibility in different adjuvanted formulations using the *S. sonnei* OPS-IpaB antigen as a model, since the competitive ELISA assay was shown to be stability indicating. When the antigen was bound to AP or AH and incubated at 25 °C for 2 weeks, we observed (1) increased antigenicity values suggesting such antigen–adjuvant interactions increased the antigen’s epitope exposure, and (2) improved stability profiles compared to antigen formulations without aluminum salt adjuvants. Specific aluminum salt + CpG adjuvanted formulations of *S. sonnei* OPS-IpaB antigen were identified (e.g., optimized levels of phosphate buffer, detergent and solution pH) and shown to be stable when stored at 2 to 8 °C for several weeks. For CpG adjuvant—aluminum salt adjuvant compatibility studies, we evaluated binding interactions and possible molecular binding mechanisms and showed that AP + CpG formulations were unstable due to the precipitation of unbound CpG interacting with the detergent LDAO (from the bulk antigen solution). In contrast, AH + CpG formulations were stable since CpG was bound to AH (likely limiting interactions with LDAO and avoiding precipitation).

In summary, an AH + CpG adjuvanted preparation of the monovalent *S. sonnei* OPS-IpaB antigen at pH 7.0 was identified as a promising formulation for future testing of an adjuvanted, quadrivalent *Shigella* OPS-IpaB antigen formulation including (1) in vivo animal immunogenicity studies to determine the effect of adjuvant type/amounts and antigen levels/dosing on the immunogenicity profiles of the each of the four *Shigella* OPS-IpaB antigens, (2) long-term storage stability studies to determine stability profiles of each glycoconjugate antigens (and the CpG adjuvant) at different temperatures, and (3) cost analysis and modeling to confirm feasibility of a low-cost formulation of this vaccine candidate is possible for use in LMICs.

## Figures and Tables

**Table 1:** Summary of binding interactions of *S. sonnei* O-PS-IpaB antigen and CpG adjuvant to the surface of the colloidal suspensions of aluminum-hydroxide adjuvant (Alhydrogel™, AH) under different solution and formulation conditions.

Panel A	Net Charge		CpG Bound to AH? Comment		Antigen Bound to AH? Comment	
	AH	Antigen				
2 mM PO_4_, pH 7	+	−			yes	Consistent with electrostatic interactions
2 mM PO_4_, pH 7, CpG	− −	−	yes	Electrostatic interaction with negatively charged CpG or ligand exchange	yes	Antigen binds with CpG bound, consistent with other molecular interactions
2 mM PO_4_, pH 5.8	+	+			yes	Consistent with other molecular interactions
2 mM PO_4_, pH 5.8, CpG	− −	+	yes	Electrostatic interaction with negatively charged CpG or ligand exchange	yes	Antigen binds AH with CpG bound—consistent with electrostatic interactions
**Panel B**	**Net Charge**		**CpG Bound to AH?** **Comment**		**Antigen Bound to AH?** **Comment**	
	**AH**	**Antigen**				
200 mM PO_4_, pH 7	−	−			no	Not bound- consistent with electrostatic repulsion
200 mM PO_4_, pH 7, CpG	− − −	−	Partial 60%- CpG mass balance loss		no	Mostly not bound (80% unbound)- consistent with electrostatic repulsion with some limited other molecular interaction mechanisms
200 mM PO_4_, pH 5.8	−	+			yes	Consistent with electrostatic interactions
200 mM PO_4_, pH 5.8, CpG	− − −	+	Partial 60%- CpG mass balance loss		yes	Consistent with electrostatic interactions

“+” indicates a net positive charge, “−” slightly negative, “− −” moderately negative, and “− − −” highly negative.

**Table 2:** Summary of binding interactions of *S. sonnei* O-PS-IpaB antigen and CpG adjuvant to the surface of the colloidal suspensions of aluminum-phosphate adjuvant (Adju-phos™, AP) under different solution and formulation conditions.

Panel A	Net Charge		CpG Bound to AP? Comment	Antigen Bound to AP? Comment	
	AP	Antigen			
2 mM PO_4_, pH 7	−	−		yes	Consistent with other molecular interactions
2 mM PO_4_, pH 7, CpG	− −	−	Partial 30%- CpG mass balance loss	yes (80%)	Mostly bound- consistent with other molecular interactions, with unbound (20%) potentially from displacement by CpG-LDAO complexes?
2 mM PO_4_, pH 5.8	−	+		yes	Consistent with electrostatic interactions
2 mM PO_4_, pH 5.8, CpG	− −	+	Partial 10%- CpG mass balance loss	yes	Consistent with electrostatic interactions
**Panel B**	**Net Charge**		**CpG Bound to AP?** **Comment**	**Antigen Bound to AP?** **Comment**	
	**AP**	**Antigen**			
200 mM PO_4_, pH 7	−	−		no	Not bound- consistent with electrostatic repulsion
200 mM PO_4_, pH 7, CpG	− − −	−	Partial 40%- CpG mass balance loss	no	Not bound- consistent with electrostatic repulsion
200 mM PO_4_, pH 5.8	−	+		yes	Consistent with electrostatic interactions
200 mM PO_4_, pH 5.8, CpG	− − −	+	Partial 40%- CpG mass balance loss	no (80%)	Bound part (20%) is consistent with electrostatic interactions, with unbound (80%) potentially from displacement by CpG-LDAO complexes?

“+” indicates a net positive charge, “−” slightly negative, “− −” moderately negative, and “− − −” highly negative.

## Data Availability

The datasets generated and/or analyzed as well as Supplementary Materials are available in the KU ScholarWorks repository: https://doi.org/10.17161/1808.36162.

## Supplementary Materials

The following supporting information can be downloaded at: https://www.mdpi.com/article/10.3390/vaccines14010010/s1, Figure S1: Measuring pI values of *S. sonnei* O-PS-IpaB conjugate antigen using cation exchange spin columns. (A) Experimental outline for use of mini spin columns for pI determination. (B) Amount (µg) conjugate antigen eluted through the column matrix over a range of solution pH values. Error bars denote SD from n = 4 (two vials analyzed at n = 2). See Section 2.2 for experimental details including the adaptation of the method protocol for pI determination from ThermoFischer. (https://www.thermofisher.com/order/catalog/product/90008 Accessed on 25 March 2025); Figure S2: Langmuir binding isotherm analysis for interaction of *S. sonnei* O-PS-IpaB conjugate antigen with the other three aluminum salt formulations (AH+CpG, AP, and AP+CpG). Calculated average values of binding capacities (*Q_max_*) and strength of interaction (*K_L_*) values are shown. (A) AH at pH 7.0 with CpG, (B) AP at pH 5.8, (C) AP at pH 5.8 with CpG. Formulations contained 2 mM sodium phosphate buffer, 0.15 M NaCl, 0.05% w/v LDAO at indicated pH values. See Figure 7 in main text for results with AH at pH 7.0. Error bars denoted range or SD from n = 2–4 (1–2 vials analyzed at n = 2). AH—Alhydrogel*®*, AP—Adju-phos*®*, and CpG—CpG 1018^®^; Figure S3: The CpG adjuvant concentration for F3, F6, F9, F12 formulations of the *S. sonnei* O-PS-IpaB conjugate antigen at time zero of the stability study. CpG concentration in the supernatant was determined by absorbance values at 260 nm (see Section 2.2.4). Composition of each formulation is described in Figure 9A of main text. Error bars denote range from n = 2 (1 vial analyzed at n = 2). AH—Alhydrogel^®^, AP—Adju-phos*®*, and CpG—CpG 1018^®^.

## Abbreviations

L-Rha	L-Rhamnose (6-deoxy-L-mannose)
D-Glc	D-Glucose
D-GlcNAc	2-Acetamido-2-deoxy-D-glucose
D-GalNAc	2-Acetamido-2-deoxy-D-galactose
L-AltNAcA	2-Acetamido-2-deoxy-L-alturonic acid
D-FucNAc4N	2-Acetamido-4-amino-2,4-dideoxy-D-fucose
D-GalA	D-Galacturonic acid
Ac	O-acetyl
O-PS	O-antigen polysaccharide
*S. f. 2a*	*Shigella flexneri 2a*
*S. f. 3a*	*Shigella flexneri 3a*
*S. f. 6*	*Shigella flexneri 6*
*S. sonnei*	*Shigella sonnei*
LMIC	Low-and-middle-income countries
WHO	World Health Organization
EED	Environmental enteric dysfunction
WRAIR	Walter Reed Army Institute of Research
AH	Alhydrogel^®^
AP	Adju-phos^®^
CpG	CpG 1018^®^
