# Supplementary material for: Compatibility and Stability of a Shigella Polysaccharide—Protein Conjugate Antigen Formulated with Aluminum Salt and CpG 1018® Adjuvants
Source: Vaccines (Basel). 2025 Dec 20;14(1):10. doi: 10.3390/vaccines14010010 (PMC12846156; doi:10.3390/vaccines14010010)
Supplement: Supplementary file 1 [file vaccines-14-00010-s001.zip › vaccines-3985862-supplementary.pdf]

Compatibility and stability of a *Shigella* polysaccharide-protein conjugate antigen formulated with aluminum-salt and CpG 1018® adjuvants

Supplemental Section

Poorva Taskar [1], Prashant Kumar [1], Brandy Dotson [1,†], Anup Datta [2], Shangdong Guo [2], Giriraj Chalke [2], Richa Puri [2], Harshita Seth [2], Benjamin Wizel [3], Sangeeta B. Joshi [1] and David B. Volkin [1,*]

[1] Vaccine Analytics and Formulation Center, Department of Pharmaceutical Chemistry, University of Kansas, Lawrence, KS 66047, USA
[2] Inventprise Inc., Redmond, WA 98052, USA
[3] Dynavax Technologies Corporation, 2929 Seventh Street, Suite 100, Berkeley, CA 94710, USA
[*] Correspondence: volkin@ku.edu; Tel.: +1-785-864-6262
[†] Current address: Mayo Clinic, 4165 Highway 14 West, Suite 300, Rochester, MN 55901, USA.

Keywords-: *Shigella* vaccine, polysaccharide-protein conjugate vaccine, compatibility, stability, formulation, adjuvant, aluminum-salt, CpG 1018, ELISA

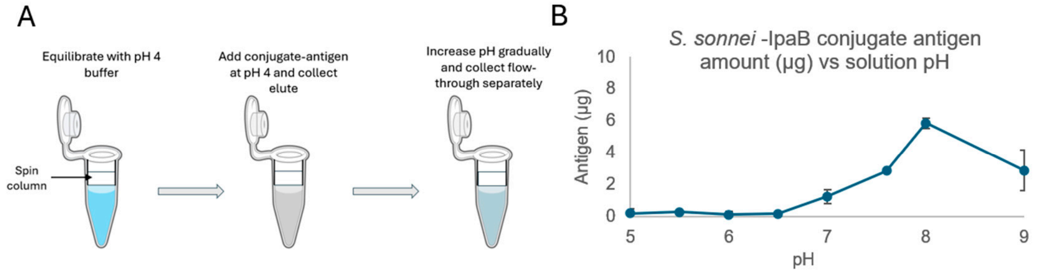

Suppl. Figure S1: **Measuring pI values of *S. sonnei O-PS-IpaB* conjugate antigen using cation exchange spin columns.** (A) experimental outline for use of mini spin columns for pI determination. (B) Amount (µg) conjugate antigen eluted through the column matrix over a range of solution pH values. Error bars denote SD from n=4 (2 vials

analyzed at n=2). See methods section for experimental details including the adaptation of the method protocol for pI determination from ThermoFischer. (https://www.thermofisher.com/order/catalog/product/90008)

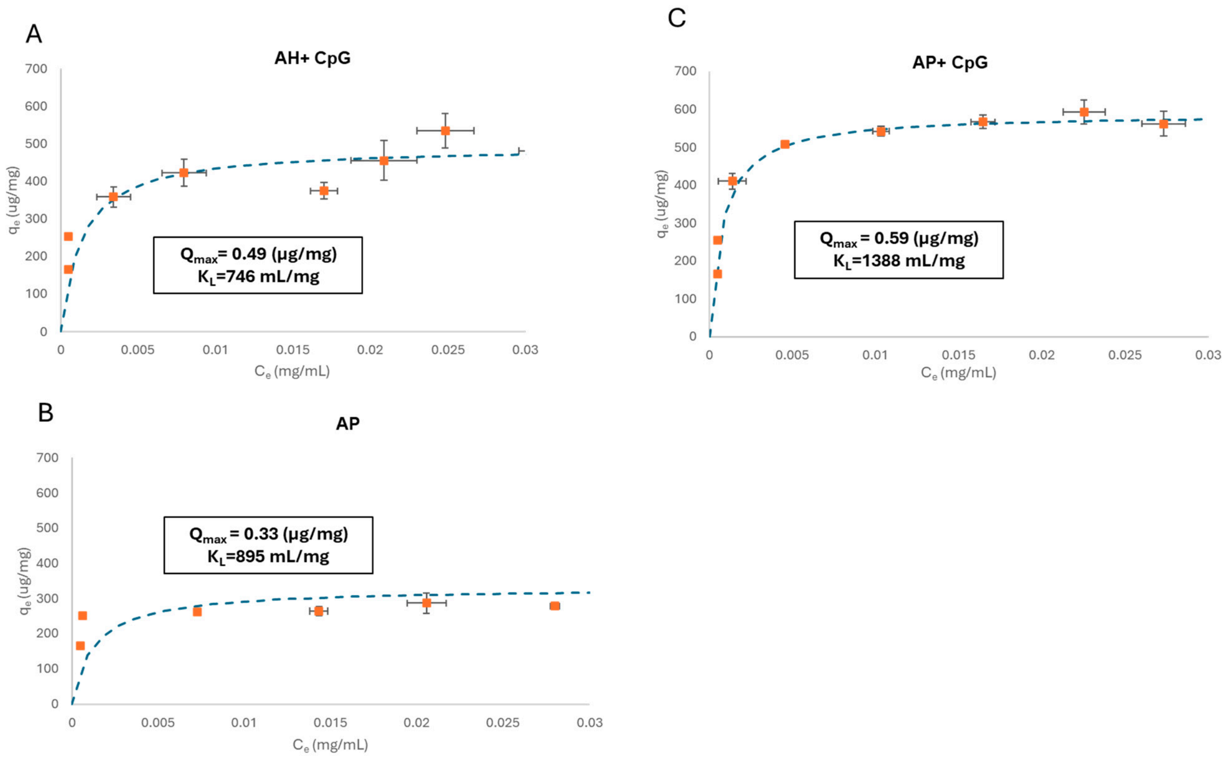

Suppl. Figure S2: **Langmuir binding isotherm analysis for interaction of *S. sonnei O-PS-IpaB* conjugate antigen with the other three aluminum-salt formulations (AH+CpG, AP, and AP+CpG)**. Calculated average values of binding capacities (Q_{max}) and strength of interaction (K_L) values are shown. (A) AH at pH 7.0 with CpG, (B) AP at pH 5.8, (C) AP at pH 5.8 with CpG. Formulations contained 2 mM sodium phosphate buffer, 0.15 M NaCl, 0.05% w/v LDAO at indicated pH values. See Figure 7 in main text for results with AH at pH 7.0. Error bars denoted range or SD from n = 2-4 (1-2 vials analyzed at n = 2). AH- Alhydrogel®, AP- Adju-phos® and CpG- CpG 1018®.

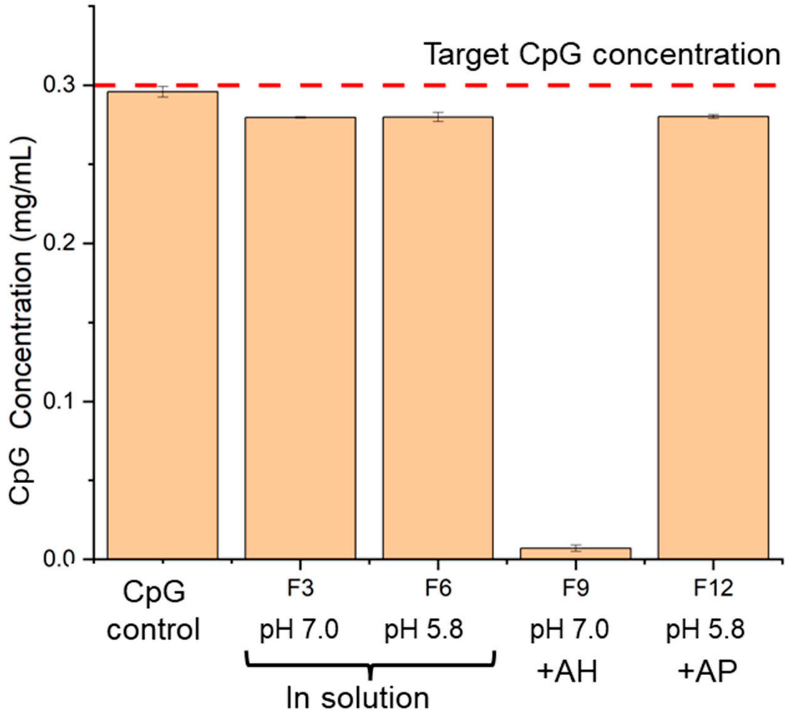

Suppl Figure S3: **The CpG adjuvant concentration for F3, F6, F9, F12 formulations of the *S.sonnei O-PS-IpaB* conjugate antigen at time zero of the stability study.** CpG concentration in the supernatant was determined by absorbance values at 260nm (see methods). Composition of each formulation is described in Figure 9A of main text. Error bars denote range from n = 2 (1 vial analyzed at n = 2). AH- Alhydrogel®, AP- Adju-phos® and CpG- CpG 1018®.
